# Have genetic targets for faecal pollution diagnostics and source tracking revolutionized water quality analysis yet?

**DOI:** 10.1093/femsre/fuad028

**Published:** 2023-06-07

**Authors:** Katalin Demeter, Rita Linke, Elisenda Ballesté, Georg Reischer, René E Mayer, Julia Vierheilig, Claudia Kolm, Margaret E Stevenson, Julia Derx, Alexander K T Kirschner, Regina Sommer, Orin C Shanks, Anicet R Blanch, Joan B Rose, Warish Ahmed, Andreas H Farnleitner

**Affiliations:** Institute of Chemical, Environmental and Bioscience Engineering 166, TU Wien, Gumpendorferstrasse 1A, 1060 Vienna, Austria; Institute of Chemical, Environmental and Bioscience Engineering 166, TU Wien, Gumpendorferstrasse 1A, 1060 Vienna, Austria; Departament de Genètica, Microbiologia I Estadística, Universitat de Barcelona, Av/ Diagonal 643, Barcelona, Spain; Institute of Chemical, Environmental and Bioscience Engineering 166, TU Wien, Gumpendorferstrasse 1A, 1060 Vienna, Austria; Institute of Chemical, Environmental and Bioscience Engineering 166, TU Wien, Gumpendorferstrasse 1A, 1060 Vienna, Austria; Division Water Quality and Health, Department of Pharmacology, Physiology and Microbiology, Karl Landsteiner University of Health Sciences, Dr.-Karl-Dorrek-Straße 30, 3500 Krems an der Donau,Austria; Institute of Water Quality and Resource Management , TU Wien, Lilienthalgasse 21 OD, 1030 ViennaAustria; Division Water Quality and Health, Department of Pharmacology, Physiology and Microbiology, Karl Landsteiner University of Health Sciences, Dr.-Karl-Dorrek-Straße 30, 3500 Krems an der Donau,Austria; Institute of Hydraulic Engineering and Water Resources Management, TU Wien, Karlsplatz 13, 1040 Vienna, Austria; Institute of Hydraulic Engineering and Water Resources Management, TU Wien, Karlsplatz 13, 1040 Vienna, Austria; Division Water Quality and Health, Department of Pharmacology, Physiology and Microbiology, Karl Landsteiner University of Health Sciences, Dr.-Karl-Dorrek-Straße 30, 3500 Krems an der Donau,Austria; Institute for Hygiene and Applied Immunology, CePII, Medical University of Vienna, Kinderspitalgasse 15, A-1090 Vienna, Austria; Institute for Hygiene and Applied Immunology, CePII, Medical University of Vienna, Kinderspitalgasse 15, A-1090 Vienna, Austria; U.S. Environmental Protection Agency, Office of Research and Development, Cincinnati, USA; Departament de Genètica, Microbiologia I Estadística, Universitat de Barcelona, Av/ Diagonal 643, Barcelona, Spain; College of Agriculture and Natural Resources, Michigan State University, East Lansing, USA; CSIRO Environment, Ecosciences Precinct, 41 Boggo Rd, Dutton Park, QLD 4102, Australia; Institute of Chemical, Environmental and Bioscience Engineering 166, TU Wien, Gumpendorferstrasse 1A, 1060 Vienna, Austria; Division Water Quality and Health, Department of Pharmacology, Physiology and Microbiology, Karl Landsteiner University of Health Sciences, Dr.-Karl-Dorrek-Straße 30, 3500 Krems an der Donau,Austria

**Keywords:** faecal pollution microbiology, DNA/RNA analysis, faecal indicator, faecal and MST markers, microbial source tracking, systematic review

## Abstract

The impacts of nucleic acid-based methods - such as PCR and sequencing - to detect and analyze indicators, genetic markers or molecular signatures of microbial faecal pollution in health-related water quality research were assessed by rigorous literature analysis. A wide range of application areas and study designs has been identified since the first application more than 30 years ago (>1100 publications). Given the consistency of methods and assessment types, we suggest defining this emerging part of science as a *new discipline: genetic faecal pollution diagnostics (GFPD)* in health-related microbial water quality analysis. Undoubtedly, GFPD has already revolutionized faecal pollution detection (i.e., traditional or alternative general faecal indicator/marker analysis) and microbial source tracking (i.e., host-associated faecal indicator/marker analysis), the current core applications. GFPD is also expanding to many other research areas, including infection and health risk assessment, evaluation of microbial water treatment, and support of wastewater surveillance. In addition, storage of DNA extracts allows for biobanking, which opens up new perspectives. The tools of GFPD can be combined with cultivation-based standardized faecal indicator enumeration, pathogen detection, and various environmental data types, in an integrated data analysis approach. This comprehensive meta-analysis provides the scientific status quo of this field, including trend analyses and literature statistics, outlining identified application areas, and discusses the benefits and challenges of nucleic acid-based analysis in GFPD.

## Teaser

The proposed discipline of genetic faecal pollution diagnostics helps addressing complex water, sanitation, and human health-related issues: this systematic review elucidates application areas, key research questions and study designs from over 1100 scientific articles and provides critical insights and conclusions.

Abbreviations 16S AmpSeq
: 16S rRNA gene amplicon sequencing
aLOD
: assay limit of detection
AMR
: antimicrobial resistance
ARB
: antibiotic resistant bacteria
ARG
: antibiotic resistance gene
CSO
: combined sewer overflow
DGGE
: denaturing gradient gel electrophoresis
dPCR
: digital polymerase chain reaction
ET-qPCR
: enzymatic treatment qPCR
FIO
: faecal indicator organism
GFPD
: genetic faecal pollution diagnostics
HAdV
: human adenovirus
HDA
: helicase dependent amplification
HRWM
: health-related water microbiology
HTS
: high-throughput sequencing
LAMP
: loop-mediated isothermal amplification
MST
: microbial source tracking
mtDNA
: (host) mitochondrial DNA
PCR
: polymerase chain reaction
PMA
: propidium monoazide
PMMoV
: pepper mild mottle virus
QMRA
: quantitative microbial risk assessment
sLOD
: sample limit of detection
USEPA
: United States Environmental Protection Agency
qPCR
: quantitative polymerase chain reaction
WASH
: water, sanitation, and hygiene
WCA
: whole chain analysis (sampling, processing, and analysis)
WWTP
: wastewater treatment plant

## Glossary

### General terms

**Genetic** (method, detection, target, and so on): nucleic acid-based

**Microbial source tracking (MST)**: methods to discriminate between human and various nonhuman sources of faecal contamination using microorganisms as primary diagnostic sources of information. Chemical and other parameters may provide complimentary information.

### Terms describing indicator types

**General FIO**: an intestinal microorganism whose presence in the environment indicates the presence of faecal matter (without discrimination among sources).

**Host-associated faecal indicator**: an intestinal microorganism, i.e. strongly associated with its particular host species or range of host species. Its presence provides information about the faecal pollution sources in the environment.

**Index organism**: a microorganism (often a faecal indicator) that indicates the presence of a specific intestinal pathogen or groups of intestinal pathogens.

**Risk indicator**: a microorganism (often a faecal indicator) for which the correlation to waterborne disease has been clearly demonstrated and quantified. Threshold values are then derived, where a certain concentration of the risk indicator corresponds, in the given exposure scenario, to a given health risk (rate of the selected waterborne disease).

**Treatment indicator**: a microorganism indicative of the behaviour of a certain pathogen (group) in wastewater treatment and disinfection processes.

**Transport surrogate**: a microorganism mimicking the behaviour of a certain pathogen (group) in surface and subsurface microbial fate and transport.

### Terms related to genetic methods for faecal pollution detection

**General faecal marker**: a nucleic acid target indicative of total faecal pollution (with no discrimination among sources), including the genetic detection of traditional general faecal indicators also amenable to cultivation-based enumeration (such as *E. coli*, enterococci) and of abundant intestinal obligate anaerobes [e.g. universal *Bacteroidota* (formerly *Bacteroidetes*) markers].

**Host-associated faecal marker or MST marker**: a nucleic acid target strongly associated with a particular host species or range of host species. Its presence in water provides information about the faecal pollution source(s) in the environment. Prokaryotic MST targets are often host-associated, occurring in nontargets at a lower rate. In contrast, viral MST targets can be host-specific and not detectable in nontargets.

**Genetic faecal pollution diagnostics (GFPD)**: any methodology that relies on the detection and/or quantification of nucleic acid-based targets to detect or characterize microbial faecal pollution in the broadest sense.

## Introduction

Safe drinking water, sanitation, and hygiene (WASH) are prerequisites to good health and well-being. Despite considerable global progress in recent decades, ~829 000 people still die each year from diarrheal disease, primarily through faecal–oral pathways, due to unsafe WASH practices (World Health Organisation 2019). While there is clear evidence that safely managed water resources, water supply, and adequate sanitation reduce the health risks related to water exposure and consumption (drinking, recreational activities, household exposure as well as transmission through irrigation, aquaculture, and so on), there is a constant, urgent need for more comprehensive, informative, and rapid microbiological assessment approaches to elucidate intricate WASH-related questions and to clarify complex faecal contamination issues.

For well over 100 years, faecal pollution assessment through the microbiological analysis of water has relied on the cultivation-based detection of facultative anaerobic bacterial colonizers of the animal and human gut, e.g. total coliforms, faecal coliforms, *Escherichia coli*, and intestinal enterococci. Recent advances in nucleic acid sequencing methods and bioinformatics have revealed the immense richness and diversity of the gut microbiota, opening unprecedented possibilities to develop new microbiological assessment approaches. Given the great diversity of assessment types made possible by genetic detection and analysis methods, we introduce the new term ‘*genetic faecal pollution diagnostics (GFPD)*’ to cover the entirety of this field, wherein ‘genetic’ means ‘nucleic acid-based’. For terms and definitions, please refer to the 'Glossary'.

Gut microbiotas are profoundly different from free-living microbial communities (e.g. Chen et al. 2018) across the biosphere (Ley et al. 2008). The Human Microbiome Project revealed *Bacteroidota* and *Firmicutes* to be the dominant phyla in the human gut, with substantial variability among individuals (The Human Microbiome Project Consortium 2012). The microbiome of municipal wastewater provides a community fingerprint that captures this diversity, with significantly lower community-level variability compared to individuals (Newton et al. 2015). In addition to faecal taxa, the wastewater microbiome also harbours a large proportion of wastewater infrastructure-related microorganisms (Shanks et al. 2013). The within-species variability in the human gut proves to be minor in comparison to the stark differences among other animal species, where both host phylogeny and diet are key drivers (Ley et al. 2008, Youngblut et al. 2019, Mallott and Amato 2021, Youngblut et al. 2021). In addition to the prokaryotic community, the gut also harbours a great diversity of viruses (bacteriophages, viruses of archaea, and of human cells as well as viruses transiently present in food; Liang and Bushman 2021). Novel molecular biological and genetic tools offer fascinating new ways to analyse and track faecal microorganisms or viruses in water. To date, these opportunities have only partially been exploited, and future research is poised to further the discovery and impacts of the GFPD field.

**Figure 1.**
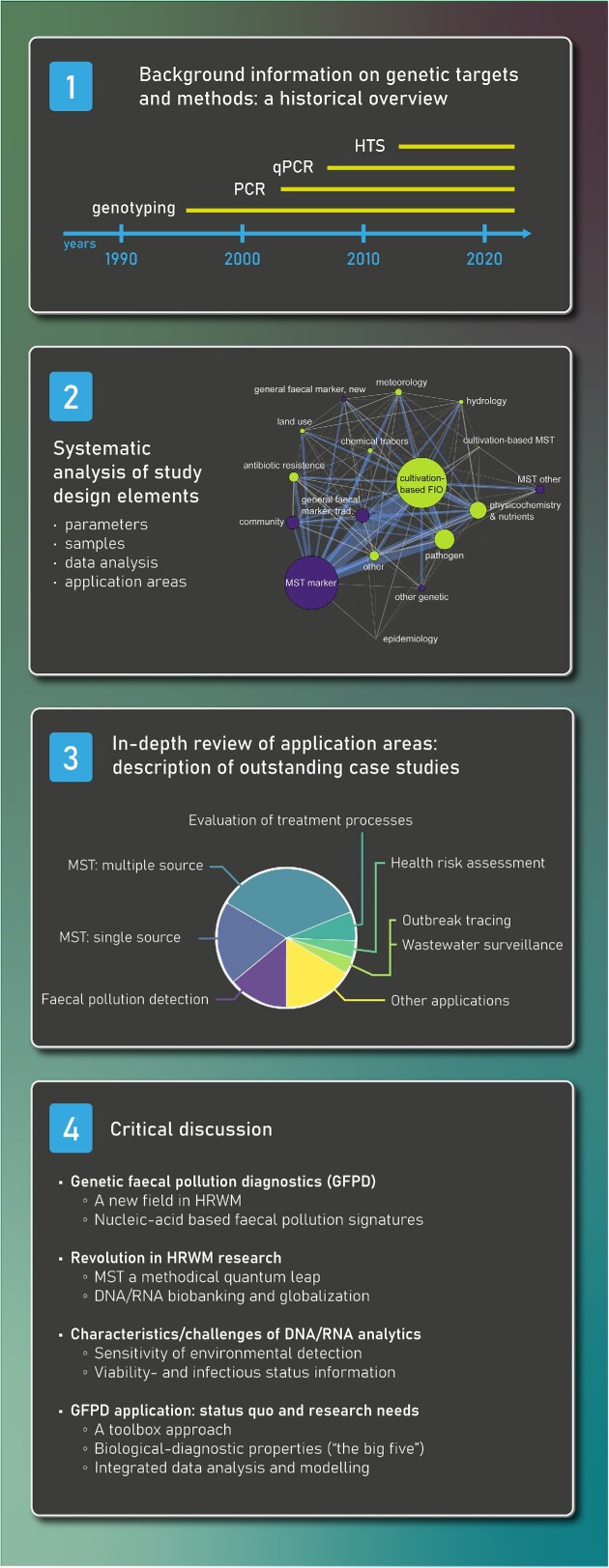
Overview and structure of this review article.

The aim of this work is to assess the impacts of nucleic acid-based methods on faecal pollution detection and analysis in the field of health-related water microbiology (HRWM). For the first time, this review provides a critical analysis of the new possibilities that state-of-the-art genetic methods have opened in a great diversity of application areas. This is accomplished via a systematic literature review to identify GFPD application areas, key research questions, and study designs from more than 1100 peer-reviewed publications, since the very beginning of using such molecular techniques in the environmental water compartment. The review focuses on genetic targets and parameters that take a faecal indication role; therefore, specific pathogen detection is only included if the indicator role is explicitly stated. Furthermore, description of the various methodological developments of molecular methods and their evaluation is outside the scope of this effort (please find a selection of methodological review articles in the section ‘*Background information on genetic targets and methods: a historical overview*’). The outcomes of the systematic literature review include trend analyses of relevant scientific literature (‘*Outcomes of the systematic study design analysis*’), followed by the analysis and discussion of seven identified application areas in HRWM (‘*In-depth review of the application areas of genetic faecal pollution diagnostic through case studies*’). The review concludes with a critical discussion on the benefits and limitations of GFPD in health-related water quality research and management. Figure 1 provides an overview of this article.

## Background information on genetic targets and methods: a historical overview

### Cultivation-based methods for faecal pollution detection: where it all began

The first routine bacteriological analyses of drinking water were initiated by Percy and Grace Frankland in London in 1885, building on the seminal work of Robert Koch and colleagues regarding microbiological media for detecting bacteria (Koch 1881). Around this time, Escherich described the bacterium that was later renamed *Escherichia coli*, in the faeces of breast-fed children (Escherich 1886, Castellani and Chalmers 1919). *E. coli* is currently one of the most widely used faecal indicator organisms (FIO; see the section ‘*Glossary*’) for water quality testing (Levine 1921, Perry and Bayliss 1936, Geldreich 1966), together with intestinal enterococci (Kjellander 1960, Geldreich and Kenner 1969) and their phages, such as somatic coliphages, and F-specific RNA bacteriophages (Grabow 2001, Jofre et al. 2016).

These standardized, cultivation-based FIO parameters have found their way into regulations all over the world and are still the gold standards for monitoring general faecal pollution in most types of water resources. While these FIOs revolutionized water quality testing and public health protection at the end of the 19th century, they also face several limitations. For example, most protocols require more than one working day to produce results, and these FIOs are unable to differentiate between faecal pollution sources (i.e. human, bird, cattle, and so on). It must be mentioned that host-associated cultivable enteric microorganisms, such as human-associated sorbitol-fermenting bifidobacteria are known (Mara and Oragui 1983, Mushi et al. 2010) and have paved the way for the field of microbial source tracking (MST; see the section ‘*Glossary*’). However, advances in molecular biology offered an unprecedented range of new opportunities to develop genetic technologies that can provide same-day water quality results and characterize key sources of faecal pollution.

### The early days of genetic methods for faecal pollution diagnostics

Faecal indicator bacteria often show tremendous genotypic subspecies variation. MST studies in the early 2000s intensively attempted to exploit this strain-level diversity by genetic fingerprinting and -typing methods (e.g. repetitive element PCR, ribotyping, amplified fragment length polymorphism, and pulsed-field gel electrophoresis) to track the origin of *E. coli* and enterococci isolates (Mott and Smith 2011). Large isolate libraries, covering faecal pollution sources and polluted water bodies in a given catchment of interest, were typed. Band/fingerprint-patterns were statistically analysed to account for the high spatial and temporal variation (*classical library-based MST*; Domingo et al. 2007, Mott and Smith 2011). Such library-based genotyping strategies were also used to evaluate the general faecal indication capacity of faecal indicator bacteria (Ishii et al. 2006, Ishii and Sadowsky 2008).

### Detection and quantification of genetic markers for faecal pollution diagnostics

Genetic characterization has led to the identification of key genes associated with a specific host, which represents a significant source of pollution (Bernhard and Field 2000). With the advent of conventional end-point PCR in the 1990s, the first studies appeared on targeted detection of general and host-associated genetic bacterial and viral targets for water quality monitoring (Bej et al. 1990, Puig et al. 1994, Bernhard and Field 2000), reviewed in Scott et al. (2002) and Noble and Weisberg (2005), which were later adapted to quantitative real-time PCR (qPCR; Seurinck et al. 2005).

The use of conventional PCR for target quantification has many limitations. Thus, qPCR appeared in the field of GFPD in the early 2000s and became the most widespread cultivation-independent technology (Jofre and Blanch 2010). Today, there are numerous qPCR assays for a wide variety of bacterial and viral targets, such as enterococci (USEPA 2012a, 2013), *E. coli* (Sivaganesan et al. 2019), human- and other animal-associated bacterial markers [original works: (Reischer et al. 2006, Shanks et al. 2008, Mieszkin et al. 2009), large-scale evaluations: (Layton et al. 2013, Reischer et al. 2013, Mayer et al. 2018), and reviews: (Wuertz et al. 2011, García-Aljaro et al. 2018)], viral MST markers including crAssphage [(García-Aljaro et al. 2017, Stachler et al. 2017) reviewed in Bivins et al. (2020)] and pepper mild mottle virus [PMMoV, (Rosario et al. 2009), reviewed in Kitajima et al. (2018), Symonds et al. (2018)] or human enteroviruses (reviewed in Farkas et al. (2020). Archaeal targets (Ufnar et al. 2006) and host mitochondrial DNA targets (Martellini et al. 2005, Schill and Mathes 2008, Malla and Haramoto 2020) have also been proposed as host-associated MST tools. Interestingly, intestinal fungi have not yet been targeted. A good overview of the most useful indicators and MST markers for which qPCR assays are available is provided in the online Global Water Pathogens Project (GWPP) book for bacterial (Harwood et al. 2018) and viral indicators of faecal pollution (Ahmed and Harwood 2017) or in a recent review article (Li et al. 2021a). Many of these methods have been subjected to multiple laboratory performance assessments and shown to be highly reproducible when standardized protocols are used (Ebentier et al. 2013, Shanks et al. 2016). Some human-associated qPCR assays are even available as government agency standardized protocols (USEPA 2019a, b) with certified companion reference materials (Kralj et al. 2021, Sivaganesan et al. 2022, Willis et al. 2022).

More recent research foci of genetic analysis methods include ease of use, rapid field-testing, and more sensitive and reproducible methods. For example, isothermal amplification assays such as LAMP (loop-mediated isothermal amplification; Martzy et al. 2017) or HDA (helicase dependent amplification; Kolm et al. 2017) have been developed for rapid enterococci detection in environmental waters; an overview can be found in Nieuwkerk et al. (2020).

In contrast to qPCR, where quantification of target genes relies on a calibration model, digital PCR (dPCR) allows quantification based on Poisson statistics of presence/absence results from thousands to millions of reaction mixture compartments per sample. Advances in microfabrication technologies in the 2010s have allowed the development of commercial dPCR platforms, making this an emerging and highly promising technology for the GFPD field (Tiwari et al. 2022).

### High-throughput DNA sequencing for genetic faecal pollution diagnostics

With the advent of high-throughput DNA sequencing (HTS) in the 2010s, whole-community profiling revolutionized gut microbiome research. This, in turn, has enabled the identification of new host-associated and general faecal pollution targets followed by the development of new qPCR assays (McLellan and Eren 2014, Bibby et al. 2019). Applying HTS to environmental samples stimulated the development of entirely new concepts for the GFPD field. HTS-based approaches have evolved rapidly, concomitant with rising capabilities in computing and bioinformatics (Garner et al. 2021). Currently, the two most widely used methods are 16S rRNA gene amplicon sequencing (16S AmpSeq) providing taxonomic information, and whole metagenome sequencing, allowing, in addition to taxonomic profiling, the identification of functional genes, such as virulence or antibiotic resistance genes (ARGs; Chan et al. 2019). There are two strategies to use HTS for faecal pollution analysis in aquatic environments. One approach works by identifying gut-associated taxa within the complex aquatic microbiome signal and thus identifying the presence of faecal pollution (e.g. Ulrich et al. 2016). The other approach relies on predefined faecal reference sequence libraries, based on a local sample collection and public sequence databases and aims to identify specific sources of faecal pollution. Sophisticated machine learning algorithms such as SourceTracker, FEAST, or FORENSIC are then required for data analysis and interpretation (Tan et al. 2015, Unno et al. 2018, Mathai et al. 2020, Raza et al. 2021). HTS, as currently applied for most applications in microbiomics, only provides relative quantification within the sequence pool recovered (% of target sequences within total recovered sequences). The resolution depends on the applied sequencing depth (i.e. number of total sequence reads per sample). Per se, it does not provide quantitative information on the analysed sequences in relation to their occurrence in the water sample (see the ‘*Sensitivity of environmental detection of nucleic acid targets*’ section). In its current form of application in GFPD, HTS seems to be of complementary nature to the qPCR/dPCR quantification of genetic fecal markers.

## Methods of the systematic study design analysis

### Literature database searches

**Figure 2.**
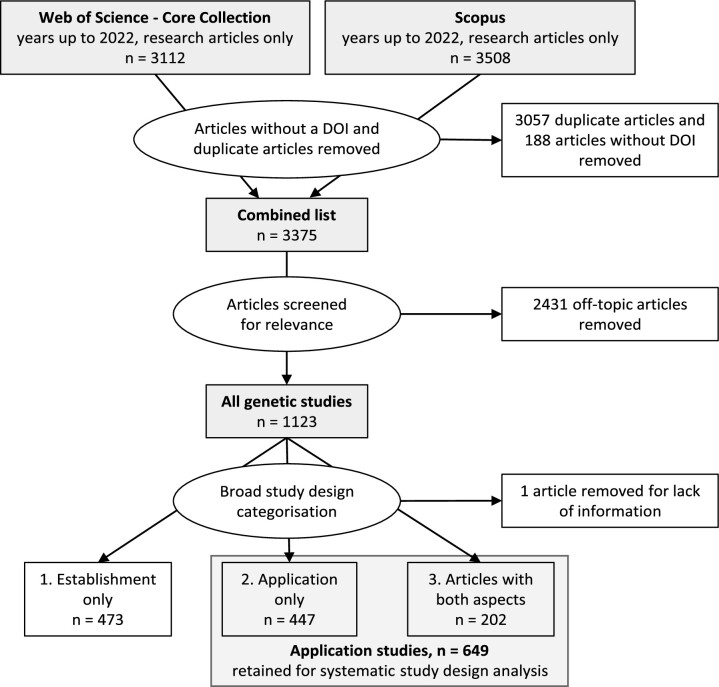
Methodology of the systematic literature analysis.

The literature databases Scopus and Web of Science/Core Collection were searched for studies on genetic methods to detect microbial faecal pollution in water. In both cases, the query included the following building blocks: ‘genetic methods’ AND ‘faeces’ AND ‘water quality’, with a suite of related words for each term. ‘Genetic methods’: (genetic OR qPCR OR ddPCR OR PCR OR ribotyp* OR DGGE OR metagenomics OR ‘microbial communit*’ OR ‘bacterial communit*’ OR ‘microbial diversity’ OR (source AND track*)); ‘faeces’: (feces OR faeces OR fecal OR faecal OR wastewater OR sewage OR enteric OR intestinal); and ‘water quality’: ((water* OR freshwater OR seawater) AND (quality OR pollution OR contamination)). Each of the blocks was searched in the title, the abstract and the author keyword fields. The document type was restricted to research articles. The time period covered expanded from the first such article up until the end of 2022. The resulting list included 3112 articles from Web of Science/Core Collection and 3508 articles from Scopus. After removing duplicates and articles with no DOI, the combined list contained 3554 articles (Fig. 2). The search syntax and the retrieved records are available as supplementary data (‘*Demeter et al GFPD review Suppl Data.xlsx*’).

### Article screening

Next, the combined list (titles and abstracts) was screened manually to remove off-topic studies. Only articles that explicitly stated the use of at least one genetic microbial parameter as an indicator for faecal pollution diagnostics (but not if used as e.g. enteric pathogen) were retained. Studies developing and evaluating new methods for GFPD as well as their field application were retained. A total of 1122 articles fulfilled these criteria (‘all genetic studies’, Fig. 2).

### Broad categorization of ‘all genetic studies’

The 1122 articles in the ‘all genetic studies’ pool were then categorized based on their *broad study aim*, as follows: (1) *method establishment* articles: the research question relates to method development and evaluation/validation (sensitivity/specificity, persistence, resistance, and so on). (2) *Application* articles: the research question relates to the environment, and the genetic parameter is assumed to have been previously validated. Studies on, e.g. the detection and source tracking of faecal pollution, or the estimation of the associated health risk, belong to this category. (3) *Both*: articles having both method establishment and application aspects. Since the review aims to assess application areas, articles from (2) and (3) were retained for detailed analysis (‘application studies’, *n* = 649, Fig. 2).

### Systematic analysis of the ‘application studies’

Titles and abstracts from all application studies (*n* = 649, Fig. 2) were reviewed to extract information on five study elements: (i) genetic faecal parameters, (ii) other types of parameters, (iii) sample type and use, (iv) data analysis approach, and (v) application area. The following section and Table 1 describe the study element definitions.

- Genetic faecal parameters: the two selection criteria for microbial parameters included here were (1) detection using genetic methods and (2) an indicator role; pathogens were only included if the indicator role was explicitly stated (e.g. ‘viral indicator’).
  - Class. Six genetic faecal parameter ‘classes’ were distinguished, where parameter ‘class’ is defined as a group of similar parameters. General faecal markers, indicating faecal pollution in general (covering human and other animal sources), are represented by two classes: ‘*traditional general faecal markers*’ that target microorganisms or bacteriophages for which the cultivation-based analysis is standardized and widely used (e.g. *E. coli*, enterococci), and ‘*new general faecal markers*’ that have been more recently developed and target highly abundant obligate anaerobes of the gut, such as Bacteroides spp. MST methods are divided into two classes: the various host-associated viral, bacterial or mitochondrial DNA-based markers are in the ‘*MST markers*’ class, while the ‘*MST other*’ class includes HTS-based as well as classical library-based, genotyping MST approaches. The class ‘c*ommunity analysis*’ covers genotyping- or HTS-based approaches to describe the microbial community. Finally, all other genetic methods for faecal pollution analysis, such as nonlibrary-based genotyping (e.g. *E. coli* population structure using strains or *E. coli* phylogroups) or treatment indicators that are not typical faecal indicators (e.g. pathogens), are included in the *‘other’* class.
  - Target organism. This study element describes taxonomical groupings covering the major target types in genetic faecal pollution analysis, such as ‘*prokaryotes*’, ‘*viruses*’, and the mitochondrial DNA of the host animal itself (‘*mtDNA’*). Other target types, such as eukaryotes using 18S rRNA gene sequencing or if the target organism was not defined, are included in the category ‘*other*’.
  - Host. Target organisms, and therefore, nucleic acid targets, may be host-associated, i.e. associated with a particular host species or narrow range of host species or may be general, i.e. associated with a wide range of host species. Four host categories are distinguished, ‘*general’*, ‘*human*’ (human- or sewage-associated), ‘*nonhuman*’ (associated with other animals), and ‘*multiple hosts*’ (more than one host was targeted). The category ‘*not applicable’* was assigned to community analyses (fingerprinting, sequencing, and so on).
  - Method. The great diversity of genetic methods for the detection of faecal pollution targets were grouped into four categories. Qualitative PCR methods (and cases where it was unclear whether qualitative or quantitative PCR was performed), are included in the category ‘*PCR*’. Quantitative PCR and dPCR are pooled because of their quantitative aspect in the category ‘*qPCR/dPCR*’. ‘*Sequencing*’ covers amplicon sequencing and whole metagenome analysis (shotgun sequencing). Finally, genetic fingerprinting techniques e.g. DGGE or BOX-PCR, hybridization, isothermal amplification, other methods or in case the method was not defined, are pooled in the category ‘*other*’.
- **Other types of parameters:**
  Class. All other parameters that the analysed articles reported were assessed on the level of parameter ‘class’, allowing an overview of the study design. Table 1 lists the 11 parameter classes that were identified. The class ‘*other*’ covers diverse parameters with low occurrence, e.g. biological oxygen demand, heterotrophic plate count, observational data on WASH practices.
- Sample type and use. A total of 13 categories of ‘sample type’, including various water types, faecal matter, and other materials, were identified. If the authors stated the intended use of the water resource, this was also logged. For a list of ‘sample type’ and ‘use type’ categories, please refer to Table 1.
- Data analysis approach. This study element describes how the dataset, characterized by the three study elements explained above, was analysed by the authors. In contrast to the three study elements, where several items could be logged, depending on the study design of the article, here each article was assigned to one of the six categories listed in Table 1. In cases in which only summary statistics were reported, we differentiated between qualitative data (occurrences) and quantitative data (minimun, maximum, median, and so on). Correlation analyses, hypothesis testing and simple bioinformatics such as sequence annotation and community analysis (e.g. Bray–Curtis dissimilarities) were grouped together into the category ‘*correlations, hypothesis tests, or simple bioinformatics*’. The category ‘*multivariate statistics or advanced bioinformatics*’ includes multivariate statistics, classification algorithms in the case of classical library-based MST, MST algorithms with HTS data, and HTS-based community analyses involving statistical analysis with metadata. Studies performing Quantitative Microbial Risk Assessment (QMRA) or microbial fate and transport models were grouped together in the category ‘*QMRA, fate & transport modelling*’. Other data analysis approaches, such as GIS-based data analysis, or, in the case of classical library-based MST, genotyping fingerprints without reporting a statistical classification method were assigned to the category ‘*other data analyses*’.
- Application area. Each article was assigned to one of the seven scientific application areas identified during the study design analysis. The application assignment is based on the predominant research question. For a list of the application areas, please refer to Table 1.

**Table 1.** Systematic study design analysis. Each article in the ‘application studies’ pool was assessed for each study element (columns), with a single or multiple choices from the categories (rows).

Genetic faecal parameters				Other types of parameters	Data analysis approach	Sample		Application area
Class	Target organism	Host	Method	Class		Sample type	Use type	
General faecal marker, traditional (e.g. *E. coli*, enterococci)	Prokaryotes	General faecal	PCR	Cultivation-based FIO	Summary statistics, qualitative data	Freshwater	Recreational	Faecal pollution detection
General faecal marker, new (e.g. general *Bacteroidetes*)	Viruses	Human or sewage	qPCR/dPCR	Cultivation-based MST	Summary statistics, quantitative data	Seawater	Irrigation	Source tracking: single source
Microbial source tracking marker (MST marker)	Host cell mitochondrial DNA (mtDNA)	Nonhuman	Sequencing	Pathogen	Correlations, hypothesis tests, or simple bioinformatics	Estuary	Drinking	Source tracking: multiple sources
Microbial source tracking, other approaches (MST other)	Other	Multiple hosts	Other	Epidemiology	Multivariate statistics or advanced bioinformatics	Domestic water	Shellfish-growing	Evaluation of treatment processes
Community analysis				Chemical tracers	QMRA, fate, and transport modelling	Groundwater	Other	Infection and health risk assessment
Other				Physicochemistry and nutrients	Other data analyses	Rainwater	N/A	Outbreak tracing and wastewater surveillance
				Antibiotic resistance		Faeces		Other applications
				Hydrology		Sewage		
				Meteorology		Stormwater, CSO		
				Land use		Soil		
				Other		Sediment and sand		
						Microcosm or spiked water		
						Other		

The assessment was performed in MS Excel. The resulting study design database (available as supplementary data, ‘*Demeter et al GFPD review Suppl Data.xlsx*’) was analysed and visualized in R, using *tidyverse* (Wickham et al. 2019). Co-occurrence networks were computed and visualized using *igraph* (Csardi and Nepusz 2006), following Ognyanova (2021). The pie diagrams over the map were created using *scatterpie* (Yu 2023) and ggplot2 (part of tidyverse). Alluvial diagrams that group and visualize categorical data, were created with *ggalluvial* (Brunson and Read 2023).

## Outcomes of the systematic study design analysis

### Broad study design trends across all articles

**Figure 3.**
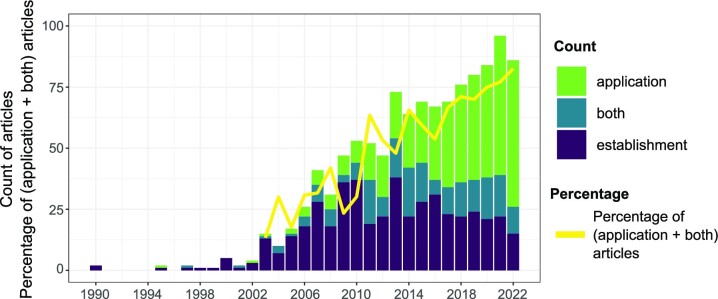
Number of publications in the broad study design types over the years in the ‘all genetic studies’ pool (*n*_article_ = 1122, Fig. 2). The stacked bars show the broad categorization. The yellow line represents the percentage of pooled ‘application’ and ‘both’ categories (i.e. the ‘application studies’, Fig. 2) in the ‘all genetic studies’ pool.

**Figure 4.**
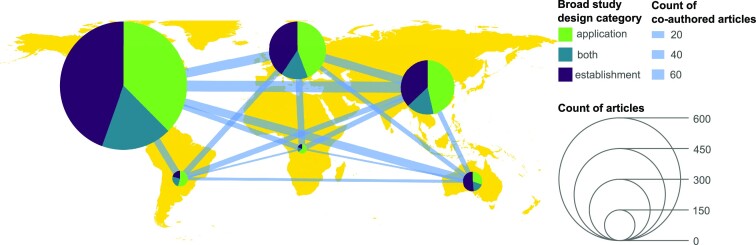
Distribution of author affiliations among continents, according to the broad study design types. The size of pies indicates the number of articles affiliated on the given continent, the thickness of lines represents the number of coauthored articles (*n*_article_ = 1122).

A systematic scientific literature database search followed by manual screening identified 1122 scientific articles (Fig. 2, ‘all genetic studies’). Research with genetic methods in this field started in the 1990s with a few articles per year, increasing to up to almost 100 articles in 2021 (Fig. 3). The broad categorization of study design types revealed three distinct phases: (i) the emergence of genetic methods in the 1990s with just a handful of articles published yearly; (ii) between ~2003 and 2010, the field started to grow with the main focus of research being on the development and validation (establishment) of new methods, namely, new general and host-associated faecal markers; (iii) since 2011, the field continues to grow, but there is a clear shift from method establishment activities to the implementation across a broad range of applications (Fig. 3). A closer look at the author affiliations reveals that Northern America is the dominant hub of both method establishment and application studies, with Europe and Asia coming second and third, respectively. Cooperation was evident among continents, demonstrating the international and interconnected nature of the GFPD field (Fig. 4).

Since the aspects of establishing methods have been duly reviewed elsewhere (see references in the ‘*Background information on genetic targets and methods: a historical overview*’ section), articles focused on these aspects were excluded from further analyses (Fig. 2).

### ‘Application studies’ trend analyses

‘Application studies’ (*n* = 649; Fig. 2) were reviewed to extract defined study elements ranging from parameters measured to ‘application area’ (Table 1, ‘*Methods of the systematic study design analysis*’). The following sections describe study element assignments and occurrence trends.

#### Parameter ‘class’ assignment and trends

**Figure 5.**
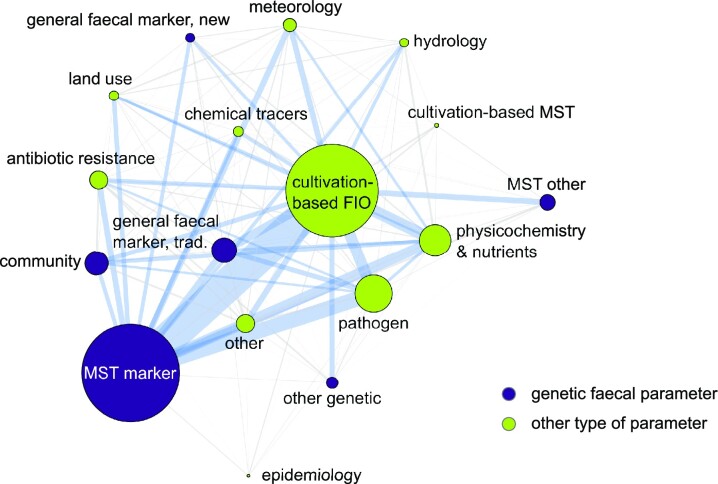
Network analysis of the parameter ‘class’ assignment occurrence in the genetic faecal and other types of parameters (Table 1) in the ‘application studies’ pool (*n*_article_ = 649). The node size is proportional to the number of articles, the line thickness reflects the number of articles for a respective combination. Blue lines mark more than 20 co-occurrences while grey lines show less than 20 co-occurrences.

Parameter ‘class’ assignments were designed to provide a coarse overview of the general experimental study design where parameter ‘class’ was defined as a group of similar parameters. A total of 17 parameter ‘class’ types, including six genetic and eleven other parameter classes, were identified during the systematic review ranging from ‘*MST markers*’ (measured by *n* = 434 articles) and ‘*cultivation-based FIOs*’ (*n* = 410) to ‘*epidemiology*’ (*n* = 13). A total of 468 articles (72% of ‘application studies’) included three or fewer parameter classes. A total of four parameter classes were reported by 116 articles, while complex study designs with five or more parameter classes were rare with only 65 articles. A co-occurrence network analysis indicated that the combination ‘*MST markers*’ and traditional ‘*cultivation-based FIO*’ was the most common one (*n* = 277 articles). In fact, not only were ‘*MST markers*’ paired often with ‘*cultivation-based FIO*’, but this was the most common combination for each of the genetic parameter classes. Additionally, ‘*MST markers*’ were often combined with ‘*pathogens*’ (*n* = 126 articles) and ‘*physicochemistry and nutrients*’ (*n* = 87 articles, Fig. 5).

#### Genetic parameters: ‘target organism’, ‘host’, and ‘method’ assignments

**Figure 6.**
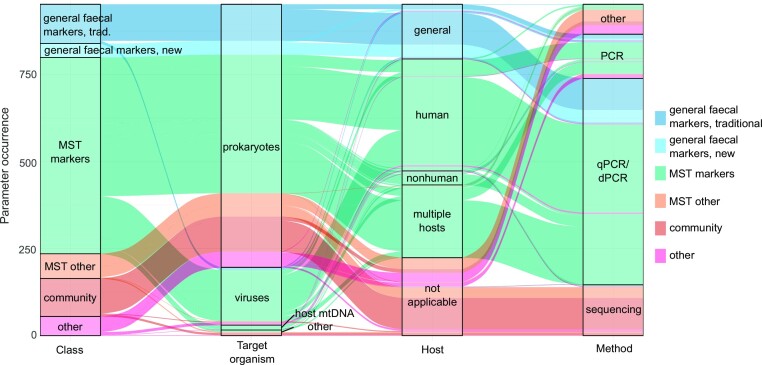
Alluvial plot showing the occurrence of genetic parameter types in the ‘application studies’ pool (*n*_article_ = 649). Each item, i.e. each line corresponds to one parameter measured in one study, so one ‘class’—‘target organism’—‘host’—‘method’ assignment. The thickness of the stratum (ribbon) corresponds to the number of studies that measured that particular class–organism–host–method combination. However, since a study might have measured several genetic parameters, the *y*-axis does not correspond to the number of articles in the ‘application studies’ pool.

All ‘application studies’ were mined for detailed information on the genetic parameters. For each parameter reported, the target organism, host organism, and analytical method were recorded, resulting in a total of 952 parameter occurrences across the 649 application studies. The most widely reported target organism was ‘*prokaryotes*’ (*n* = 756 parameter occurrences) followed by ‘*viruses*’ (*n* = 166). In contrast, ‘*host mitochondrial DNA*’ and ‘*other*’ target organisms collectively accounted for 30 parameter occurrences. Host assignments indicated that ‘*human*’ (*n* = 322) is the most widely researched host animal followed by ‘*multiple hosts*’ (*n* = 209), ‘*general*’, ‘*faecal*’ (*n* = 157), and ‘*nonhuman*’ (*n* = 40). Method assignments suggest that PCR-based methods account for the vast majority of parameter occurrences (*n* = 720), with ‘*qPCR/dPCR*’ methods used 82% of the time. ‘*Sequencing*’ was the next most prevalent method assignment group (*n* = 146). An alluvial plot (Fig. 6) illustrates linkages or lack thereof between class, target organism, host, and method parameters.

#### Sample ‘type’ and intended ‘use’ assignments and trends

**Figure 7.**
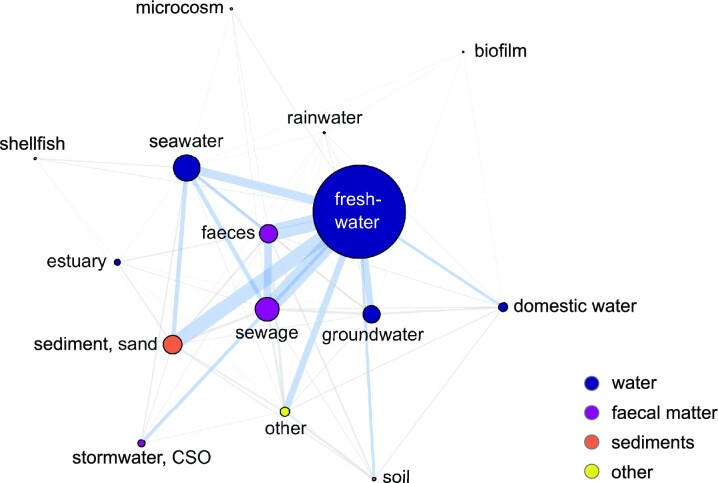
Network analysis of the ‘sample type’ assignment occurrence in the ‘application studies’ pool (*n*_article_ = 649). The node size is proportional to the number of articles, the line thickness reflects the number of articles for a respective combination. Blue lines mark more than 10 co-occurrences while grey lines show less than 10 co-occurrences. CSO denotes combined sewer overflow.

**Figure 8.**
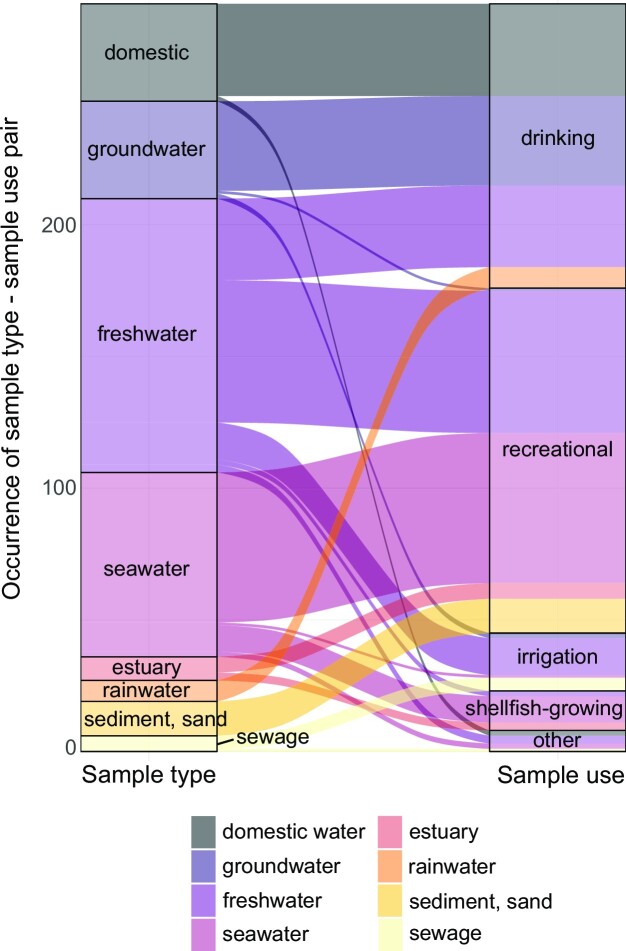
Alluvial plot showing the ‘sample type’—‘sample use’ combinations in the subpopulation of ‘application studies’ that reported this information (*n*_article_ = 254 articles). Since a study might have analysed several ‘sample types’, or indicated several ‘water uses’, the *y*-axis does not correspond to the total number of articles.

A total of 14 sample types were identified ranging from ‘*freshwater*’ (*n* = 394 articles, 61% of articles) and ‘*seawater*’ (*n* = 113) to ‘*rainwater*’, ‘*microcosm*’, ‘*shellfish*’, and ‘*biofilm*’ (each *n* ≤ 10, Fig. 7). The most common combinations were ‘*freshwater*’ and ‘*sewage*’ (*n* = 56), ‘*freshwater*’ and ‘*faecal matter*’ (*n* = 56) and ‘*freshwater*’ and ‘*sediments and sand*’ (*n* = 50, Fig. 7). Of the 836 reported sample types, where an intended use would potentially be relevant (i.e. all water types, ‘*sewage*’ and ‘*sediment and sand*’), the intended use was reported for 284 sample types, representing 254 articles. ‘*Recreational*’ and ‘*drinking*’ water were the most frequently described, accounting for 131 and 108 occurrences, respectively. ‘*Irrigation*’ and ‘*shellfish-growing*’ were seldom studied (*n* = 22, *n* = 15 occurrences, Fig. 8).

#### ‘Data analysis approach’ assignment and trends

**Figure 9.**
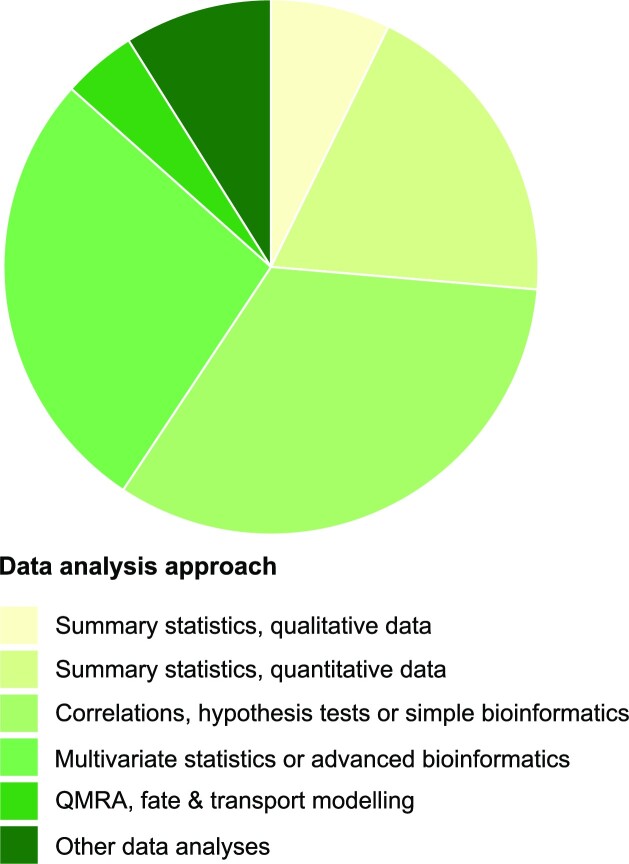
Data analysis approach in each article in the ‘application studies’ pool (*n*_article_ = 649).

While 171 articles (26%) only report summary statistics (qualitative and quantitative), the majority report more sophisticated data analysis approaches such as ‘*correlation analyses, hypothesis tests or simple bioinformatics*’ (*n* = 214, 33%) and ‘*multivariate statistics or advanced bioinformatics*’ (*n* = 177, 27%). ‘*QMRA or fate & transport modelling*’ were found to be conducted only by a small portion of the articles (*n* = 29, 4%, Fig. 9).

#### ‘Application’ type assignment and trends

**Figure 10.**
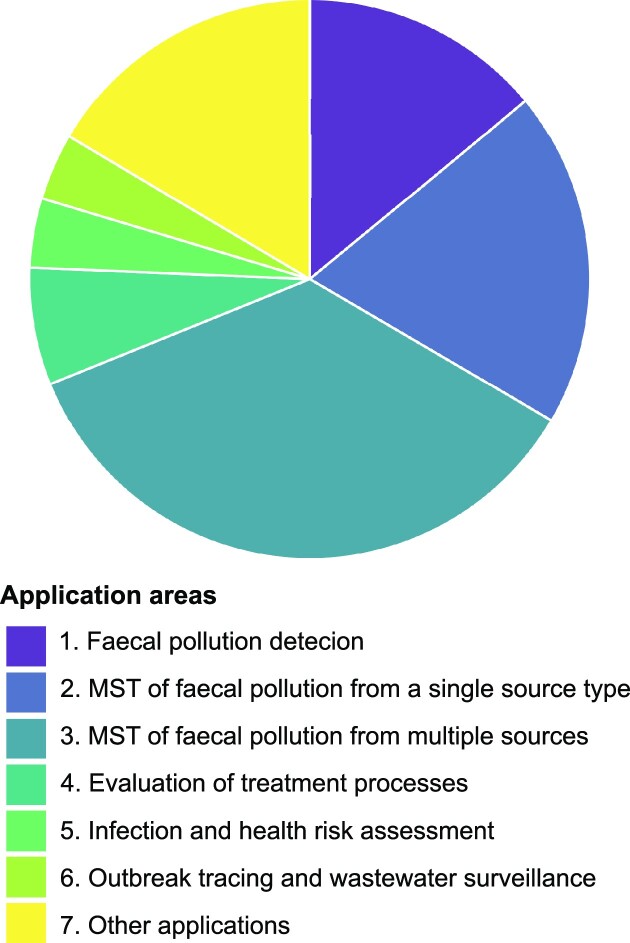
Assigned application areas in the ‘application studies’ pool (*n*_article_ = 649).

A total of seven genetic method application areas were identified in this systematic literature review (Fig. 10). In addition to faecal pollution detection using general faecal indicators (‘*Application 1*’, 91 articles), MST was the predominant use of genetic faecal markers (‘*Application 2*’ and ‘*Application 3*’, 356 articles in total). Most of these studies performed MST in the classical sense, investigating several potential sources (‘*Application 3*’, 230 articles), while 126 articles targeted just one source type, mainly human (‘*Application 2*’). To a much smaller extent, genetic faecal markers were found in performance assessments of (waste)water treatment and in studies of microorganism fate and transport in groundwater as transport surrogates (‘*Application 4*’, 44 articles). An equally small, but emerging field is health and infection risk assessment, where genetic methods have been found to be employed as risk indicators, or as support in selected steps of QMRA (‘*Application 5*’, 26 articles). Host-associated faecal indicators have also been used to trace the origin of waterborne outbreaks, elucidate pathogen transmission routes and support the interpretation of SARS-CoV-2 wastewater surveillance data (‘*Application 6*’, 25 articles). Apart from these core application areas, GFPD tools have also been found to support other scientific disciplines, such as the tracking of the source of nutrients or ARGs, as well as archaeology. The section ‘*Application 7*’ provides an overview of these additional areas (107 articles).

## In-depth review of the application areas of genetic faecal pollution diagnostic through case studies

**Figure 11.**
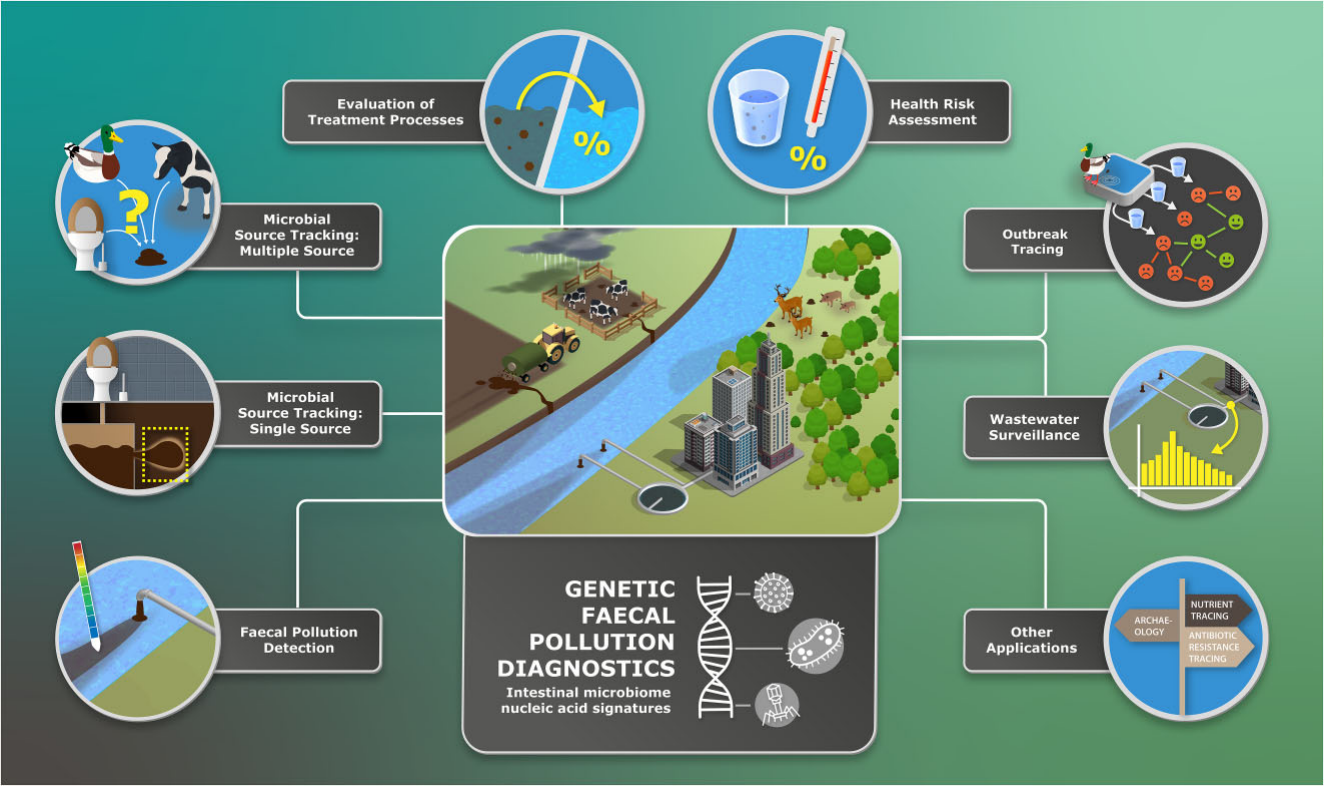
Overview of the application areas of the genetic faecal pollution diagnostics field.

The following sections demonstrate the successful implementation of GFPD in the seven identified application areas of water quality research (Fig. 11). To do so, trend analyses of selected study elements for a given application area are presented at the beginning of each section, followed by an illustration of these findings through a collection of cutting-edge case studies.

### Application 1: faecal pollution detection

In general, there are two approaches to detect faecal pollution using genetic methods, and the 91 articles in this application category can be divided along these lines, with just a small overlap: (i) the targeted detection of traditional or new general faecal markers, mostly using qPCR (for definitions, see the section ‘*Systematic analysis of the ‘application studies*’, *n* = 36 articles); (ii) the nontargeted detection of faeces-related taxa using HTS (*n* = 50); and (iii) five articles measuring both. ‘*Traditional general faecal markers*’ were used more often than ‘*new general faecal markers*’ (*n* = 37 and *n* = 9 articles, respectively). In most instances, ‘*traditional general faecal markers*’ were measured in parallel with the corresponding ‘*cultivation-based FIO*’ parameter (28 out of 37 articles). The dominant method for community composition analysis was 16S AmpSeq (45 articles). ‘*Freshwater*’, ‘*seawater*’, and ‘*sediments and sand*’ were the most common sample types while ‘*recreational*’ and ‘*drinking*’ were the most frequently observed intended use types.

#### Targeted detection of general faecal indicators

Regulatory agencies, such as the United States Environmental Protection Agency (USEPA) have begun to capitalize on the potential of qPCR as a rapid monitoring solution for recreational waters, providing same-day results (< 4 h). In 2012, water quality beach action values for qPCR measurements of enterococci were included in the U.S. Recreational Water Quality Criteria (USEPA 2012b). This addition was based upon epidemiological studies conducted at freshwater and marine beaches that provided evidence that enterococci levels measured by qPCR are predictive for swimmer-related illness ((Wade et al. 2008, 2010), see details in the section ‘*Application 5*’).

Since then, enterococci qPCR (USEPA Methods 1611 and 1609.1) has been applied in several beach monitoring demonstration and implementation programs (Ferretti et al. 2013, Dorevitch et al. 2017, Byappanahalli et al. 2018). In one of the largest studies, nine Chicago beaches were monitored over the course of 894 beach-days in 2015 and 2016, resulting in 1796 water samples that were analysed by enterococci qPCR while maintaining standard *E. coli* cultivation testing, which is typically used at the Great Lakes (Dorevitch et al. 2017). Side-by-side comparison of the two approaches showed that enterococci qPCR beach action values were exceeded 3.4 times less frequently than *E. coli* cultivation beach action values (6.6% vs. 22.6% of beach-days) (Dorevitch et al. 2017). However, generalizations—such as that qPCR testing necessarily leads to fewer beach action value exceedances than cultivation-based testing—cannot be made. Several prior studies have found varying levels of agreement between *E. coli* cultivation and enterococci qPCR beach action value exceedances (Haugland et al. 2014, Byappanahalli et al. 2018). Moreover, data analysis of this large multibeach, multiyear evaluation study found that prior-day *E. coli* cultivation results are no better than chance alone at predicting current-day water quality at Chicago beaches (Dorevitch et al. 2017). Based upon these findings, enterococci qPCR testing was expanded by the local authority at up to 20 Lake Michigan beach locations from 2017 onwards and *E. coli* cultivation-based testing was discontinued (Shrestha and Dorevitch 2020).

More recently, the USEPA developed a draft standard method for qPCR testing of *E. coli* (‘Draft Method C’; Sivaganesan et al. 2019) driven by the need for rapid *E. coli* testing. In a large-scale method comparison effort, data from 101 Michigan (USA) recreational beaches from more than 6000 samples showed 91.5% agreement in beach notification outcomes between the cultivation-based standard of 300 MPN or CFU/100 ml and a putative threshold of 1.863 log_10_ gene copies/reaction, estimated in this study (Haugland et al. 2021). A strong correlation was observed between cultivation and qPCR results, with a Pearson *R*-squared value of 0.641 for the pooled data of the 39 sites passing the data eligibility criteria (sample *n* = 2092) (Haugland et al. 2021).

The universal *Bacteroidales* marker BacUni, a new general faecal marker, was evaluated together with three cultivation-based FIOs as a predictor of protozoan and bacterial pathogens in samples from rivers and estuaries in California, USA (Schriewer et al. 2010). The universal *Bacteroidales* marker was detected in all water samples at concentrations two orders of magnitude higher than cultivation-based FIOs. The results also showed the universal *Bacteroidales* marker to have a comparable or higher mean predictive potential than cultivation-based FIOs (Schriewer et al. 2010). The high abundance of new general faecal markers is certainly an asset, as sensitivity can become a challenging aspect for genetic faecal pollution detection in water resources with low faecal pollution levels (for details, see ‘*Sensitivity of environmental detection of nucleic acid targets*’ in the ‘*Discussion*’).

#### Nontargeted detection of faeces-related taxa using high-throughput sequencing

HTS approaches have emerged in microbial water quality monitoring allowing for new opportunities. From a public health perspective, HTS surveys have been shown to identify faecal taxa (e.g. *Bacteroides*) in aquatic microbial communities (Ulrich et al. 2016, Vadde et al. 2019). For instance, Ulrich et al. (2016) tracked changes in bacterial community composition in a riverine system during and after Superstorm Sandy (a 100-year storm event in 2012) using HTS and traditional cultivation-based faecal indicator testing. Bioinformatic analyses of 16S AmpSeq data showed a drastic restructuring of the bacterial community, associated with hydrological dynamics. The relative abundances of sequences matching faecal bacteria (*Bacteroides, Clostridium*, and *Blautia* genera) and potentially pathogenic populations (*Campylobacter* and *Helicobacter*) were observed to increase after the peak of the storm (Ulrich et al. 2016). Given that HTS applications can provide profiles of microbial communities and information on faeces-associated taxa, such genetic approaches may become useful as a screening tool in the future for identifying potential health risks and for prioritizing sites for follow-up analysis of water samples using targeted quantitative PCR approaches (Vadde et al. 2019, Jiang et al. 2020).

### Application 2: MST of faecal pollution from a single source type

Faecal pollution may originate from a multitude of point and nonpoint sources. The need to identify the sources of faecal pollutions arose years ago, and since then, many different approaches have been developed and validated (‘*Background information on genetic targets and methods: a historical overview*’). Focusing the investigation on a single type of faecal source often happens (i) if there is evidence regarding the dominant source of pollution such that neglecting other sources is acceptable or (ii) the investigation specifically addresses one source type because, e.g. some faecal sources could represent a higher public health risk than others. In any case, the need to validate the hypothesis of the origin of contamination using a reliable analytical tool exists, since scientific evidence facilitates posterior effective measures.

Of the 126 articles in this application area, the single source was ‘*human*’ in 113 cases and only a handful of articles focused on ‘*nonhuman*’ sources such as ruminants, gulls, ducks, chickens, or dogs. The majority, 73%, of the articles combine ‘*MST markers*’ with the measurement of traditional ‘*cultivation-based FIOs*’. Other parameter classes that often appeared were ‘*pathogens*’, ‘*traditional general faecal markers*’, ‘*physicochemistry and nutrients*’ and ‘*chemical tracers*’ (*n* = 31 to *n* = 15 articles). ‘*Freshwater*’ was most often sampled (*n* = 80 articles), followed by ‘*seawater*’ (*n* = 23) and ‘*sewage*’ (*n* = 18). A total of 44 articles reported ‘*summary statistics*’ (qualitative or quantitative), while 48 articles performed ‘*correlations, hypothesis testing or simple bioinformatics*’. A smaller set of articles performed more advanced data analyses, such as ‘*multivariate statistics or advanced bioinformatics*’ (*n* = 23) or ‘*QMRA, fate & transport modelling*’ (*n* = 4).

#### Human sources: decentralized wastewater systems

The interpretation of MST results is greatly enhanced by cultivation-based FIO and land-use data or additional parameters that can help to explain the origin, fate and transport of a specific pollution source. For example, in watersheds with more than 1621 septic systems in Michigan, USA, higher concentrations of *Bacteroides thetaiotaomicron* (human-associated marker) were detected under baseflow conditions suggesting that control measures should include septic system maintenance and construction in the area (Verhougstraete et al. 2015). In this study, analyses were performed using a classification regression tree including riparian buffers, septic tanks, and physicochemical data. Beyond chronic pollution scenarios, rainfall events can impair water quality through combined sewer overflows, septic tank seepages, agricultural runoff or other events governed by precipitation. A similar study found that three human-associated *Bacteroides* markers correlated positively with septic tank density during wet weather, suggesting that septic tanks are a significant source (Peed et al. 2011). Since there was no correlation with FIO during baseflow conditions, the authors postulate that other sources might be implicated in chronic pollution.

#### Human sources: centralized wastewater systems

In some cases, genetic MST markers can be combined with other types of tracers to strengthen the interpretations and to overcome markers’ limitations, such as low specificity, differing decay rates or different transport. For example, the detection of the human-associated genetic marker HF183 and optical brighteners in private drinking water supplies in rural areas of Virginia, USA, revealed sewage as a potential pollution source. However, only a few samples showed *E. coli* together with the optical brighteners, suggesting a different fate and transport of these indicators within the aquifer (Smith et al. 2014). In Montreal, Canada, a study applied a multiparameter source tracking toolbox combining chemical source tracking markers for sewage (caffeine, theophylline, and carbamazepine) together with the human-associated genetic markers HF183 and mitochondrial DNA to detect illicit wastewater discharges into storm sewers during dry weather (Hachad et al. 2022). The authors used a composite index of the different markers together with the levels of *E. coli* to identify household cross connections or indirect illicit discharges and verified them successfully with dye tracing.

Hydrological and meteorological data are often indispensable to understand the fate of faecal microorganisms in the environment. For example, hydrological and meteorological data combined with the human-associated marker HMBif, cultivation-based MST parameters and FIO allowed modelling the self-depuration distance of a small Mediterranean river (Pascual-Benito et al. 2020). The obtained models provided information about the recuperation of the river’s initial conditions after receiving treated sewage discharge. MST tools are also useful after extreme meteorological events. For example, after Hurricane Harvey, the detection of the human-associated markers HF183 and BacHum and their correlation with FIO indicated a large input of sewage through sewage overflows and stormwater in two catchments in Texas, USA (Kapoor et al. 2018).

HTS applications have also been reported. After the pioneering work of Unno et al. (2010) in South Korea, the study by Newton et al. (2013) was one of the first large-scale studies that also demonstrated the complex challenges in data interpretation. The authors examined chronic human faecal pollution at an urban site in Lake Michigan, USA, and set out to identify its sources and delivery routes. By identifying the relative abundance of sewer infrastructure-associated, faecal and human faecal signatures in lake water samples, they identified combined sewer overflows as the dominant pollution source during heavy rainfall events, whereas nonhuman faecal sources exhibited the highest relative abundance during dry weather and noncombined sewer overflow producing rain events. More recently, Zimmer-Faust et al. (2021) tracked the plume of a wastewater treatment plant (WWTP) outfall in the coastal Pacific Ocean on the USA/Mexico border and showed that its behaviour differs depending on oceanic and meteorological conditions. They used a human-associated MST marker and 16S AmpSeq together with the algorithm SourceTracker, with pristine marine water, WWTP discharge and a nearby river as potential sources to derive the spatial extent and concentration gradient of human pollution.

#### Recreational waters

Coastal waters have important value for leisure, tourism, and coastal ecosystems including shellfish harvesting areas; therefore, MST tools have been extensively tested in these areas (Korajkic et al. 2009, González-Fernández et al. 2021). In Thailand, Kongprajug et al. (2021) used two genetic viral MST markers, crAssphage and HPyV, at various beaches during dry and wet seasons to verify human waste practices as the main faecal source. Their results reported temporal variability but not spatial variability, thus recommending a future monitoring strategy based on more frequent sampling at a unique sentinel site. Other studies include environmental data such as precipitation and solar radiation, oceanographic data like tides and currents, and use correlations or more complex models to be able to predict a potential pattern. For example, at different sites in San Francisco, USA, the human-associated marker HF183 was found to correlate mainly with 72 h precipitation but also water temperature, tides or insolation (Jennings et al. 2018). Cao et al. (2018) sought to develop a standardized data analysis approach that incorporates all qPCR measurements from a defined group of samples (i.e. nondetections, detections, and measurements in the range of quantification) to assess average human faecal pollution levels at recreational water sites. The authors proposed a metric, the human faecal score, that combines the results of the human-associated qPCR marker HF183/BacR287 with a defined sampling strategy (sampling intensity and number of replicates) and a Bayesian weighted average approach. The score can be used to prioritize sites for remediation and has more recently been used to compare source-associated impacts under wet and dry conditions (Shrestha et al. 2020) and identify trends with cultured FIO paired measurements (Li et al. 2021b). In addition to human sources, wild animals can also contribute to faecal indicator bacterial loads in coastal areas with large gull colonies. The presence of the gull-associated bacterium *Catellicoccus marimammalium* in 58% of the water samples and at all sampling sites as well as their correlation with faecal indicators suggested a chronic impact of gull faeces on the water quality in southern Ontario, USA (Lu et al. 2011). The same marker showed a decrease together with faecal indicators and bacterial pathogens after gull removal in Lake Michigan, USA (Converse et al. 2012).

#### Rural areas, domestic animals

Single source characterization is also relevant in rural areas with high agricultural pressure where tracking animals such as swine, ruminants, or poultry can be of interest (Weidhaas et al. 2011, Heaney et al. 2015, Wiesner-Friedman et al. 2021). These studies include, in addition to the relevant genetic faecal marker, data on land uses, land-applied manure, and/or animal feeding operations. For example, after testing for a ruminant-associated marker, BoBac, and including data on animal feeding operations, the authors found that applying manure in the fields implied an increase in faecal indicators in riverbed sediments (Wiesner-Friedman et al. 2021).

### Application 3: MST of faecal pollution from multiple sources

Many impaired water bodies are polluted by more than one source. Thus, it is important to characterize key sources because the corresponding health risk as well as the mitigation steps may differ by source. Nevertheless, study design and choice of methods are highly dependent on the water resource type, the intended water use and other factors.

Of the 230 articles with a focus on multisource MST, MST was achieved predominantly using ‘*MST markers*’ (*n* = 180, 78% of articles) followed by classical library-based MST (*n* = 33, mostly published before 2015) or HTS (*n* = 21, mostly published after 2015). In multisource MST articles, FIOs are measured predominantly with cultivation-based methods (‘*cultivation-based FIO*’, *n* = 163 articles). In contrast, ‘*traditional*’ and ‘*new genetic faecal markers*’ played a minor role (*n* = 17 and *n* = 20, respectively). The most common parameter combination was ‘*MST markers*’ with ‘*cultivation-based FIO*’ (*n* = 132, 57% of articles). Other common parameter classes were ‘*physicochemistry and nutrients*’, ‘*pathogens*’, ‘*meteorology*’, and ‘*land use*’ (*n* = 50 to *n* = 25 articles). The proportion of articles with four or more parameter classes was higher than in single-source MST (31% in multisource MST and 28% in single-source MST, ‘*Application 2*’). This higher study design complexity was reflected in the data analysis approach: 35% of articles performed ‘*multivariate statistics or advanced bioinformatics analyses*’ (18% in single-source MST, ‘*Application 2*’).

#### Elevated pollution levels on a watershed scale

The starting point in watershed studies is usually elevated levels of cultivation-based FIOs in rivers, lakes, or coastal waters. Often, the spatial scale is relatively large and there are multiple potential sources ranging from human faeces (via leaky infrastructure, treated, or untreated wastewater or combined sewer overflows) to livestock (grazing or stabled), pets as well as avian and mammalian wildlife. Often, there is limited knowledge on hydrology, meteorology, and land use. An illustrative example is given by three studies conducted over a span of 16 years in the Tillamook Bay catchment in Oregon, USA demonstrating how state-of-the-art genetic MST applications have evolved over time. Bernhard et al. (2003) and Shanks et al. (2006) compared PCR-based ruminant and human marker frequencies with faecal pollution levels considering rainfall patterns and seasonal pollution dynamics to identify pollution sources. Much more recently, Li et al. (2019) used quality-controlled and, in several cases, standardized qPCR assays for five faecal sources, and high-resolution GIS for land-use and meteorological data to not only identify but also quantify and locate pollution sources and patterns to guide remediation efforts and risk assessment. In a similar approach, Bushon et al. (2017) ranked tributaries to the Little Blue River catchment in Missouri, USA, based on estimated contributions to water quality impairment. The studies by Nguyen et al. (2018) and Yamahara et al. (2020) demonstrate how hypothesis-formulation can support study design for GFPD. Both studies also try to shed light on the potentially confounding role that soil and sediments might have on MST applications, especially in tropical waters. To elucidate the relative roles of human and other animal sources polluting the Danube River and its tributaries, Kirschner et al. (2017) used a combination of longitudinal survey along more than 2500 km of river and a temporal survey over the course of a year at three sites successfully identifying human waste as the dominant source. Bambic et al. (2015) encountered difficulties segregating pollution sources due to the confounding influence of disinfected municipal wastewater. Separating wet from dry weather based on meteorological data allowed data interpretation, with municipal wastewater (human) being the dominant dry-weather pollution source, while during wet weather, agricultural runoff, and stormwater (ruminant and dog) dominate. Using bacterial and viral markers allowed the authors to demonstrate the difficulty to detect the presence of viral pathogens when only using bacterial indicators. The authors used cutting edge data handling methods, including statistical methods to account for the large proportion of nondetects, and an estimation of spatial and temporal variations of same-host contribution using ratios between given *Bacteroidales* MST markers and a general *Bacteroidales* marker (Bambic et al. 2015). Separating the sample set into dry and wet periods allowed Liang et al. (2021) to reveal differing pollution pathways. The results of MST markers agreed with those from 16S AmpSeq and the FEAST algorithm: humans were the main pollution source in the dry season, and ruminant and swine were the main pollution sources in the wet season at this river site near Beijing, China. MST methods have also been used to more generally identify factors and features that promote or reduce watershed faecal pollution rather than just identifying pollution sources. As an example, Green et al. (2021) used MST and cultivation-based FIO in an investigation of 68 streams in New York State, USA, to identify stream features, land use practices and meteorological patterns that drive faecal pollution levels from multiple sources.

#### Recreational waters

In contrast to general watershed pollution scenarios, bathing water studies are usually triggered by persistently elevated FIO levels at public beaches directly threatening the health of visitors, necessitating beach closures and inflicting considerable economic damage. Study areas are often smaller, and the potential sources are less diverse (e.g. sewage discharges, birds, and pets) (Staley et al. 2018). Prudently, studies often make efforts to consider the influence of hydrology (flows, tides, and so on) and the effect of precipitation and solar radiation on water quality changes and to resolve faecal source contributions (Williams et al. 2022). In a proof of concept study in Xiamen, China, An et al. (2020) used high-throughput qPCR for a large number of assays targeting multiple faecal sources and pathogens to investigate bathing waters.

#### Drinking water

Impairment of drinking water quality is one of the most pressing issues worldwide. The specific challenge in this application field is that low levels of pollution already pose relevant health risks. For example, elevated FIO levels observed in karst and fractured aquifers after precipitation were the starting point for several MST studies. The problem of highly variable pollution dynamics in the course of very short time periods can be approached by linking sampling to hydrological dynamics (Reischer et al. 2008) and nested sampling with higher sampling frequencies during periods of hydrological fluctuations and during/after rainfall events (Reischer et al. 2011). The very short residence times of faecal pollution in the studied springs also allowed direct source apportionment based on MST marker concentrations in spring water because differential persistence can be disregarded when measuring very recent pollution. To determine the source and risk factors for nitrate and microbial pollution in private dolomite karst wells, Borchardt et al. (2021) used multivariate regression models with potential drivers such as land use, precipitation, hydrogeology, and well construction.

#### Aquaculture and irrigation water

Shellfish harvesting areas in coastal waters and aquaculture in general are also under a large amount of anthropogenic pressure often resulting in the contamination of products with FIOs and pathogens. The applicability of MST approaches to identify and prioritize pollution sources has been demonstrated in shellfish harvesting waters and products such as oysters (Mieszkin et al. 2013). Klase et al. (2019) integrated MST markers, ARG assays, and pathogen detection with bacterial community-based analysis to broadly investigate the potential public health risks associated with pollution of fishponds. Similarly, faecal pollution levels, ARG and pathogen occurrence were investigated in irrigation waters used for fresh produce to determine sources of pollution and risk factors (Weller et al. 2020).

### Application 4: evaluation of treatment processes

Pathogen removal is one of the primary functions of wastewater and drinking water treatment. However, relying on direct pathogen determination only is not practicable due to the low and varying concentrations in raw water as well as the high number of different pathogens potentially occurring. Thus, treatment performance assessment often relies on treatment indicators used as representative surrogates for pathogen removal (see the section ‘Glossary’; Momba et al. 2019). While cultivation-based microbial parameters are the most commonly employed treatment indicators (Jofre et al. 2016, Momba et al. 2019), the systematic literature review revealed 44 articles that used genetic markers as treatment indicators. In this article pool, ‘*MST markers*’ and ‘*traditional general faecal markers*’ were the most often measured genetic parameter classes (23 and 18 articles), whereas ‘*cultivation-based FIO*’ and ‘*pathogens*’ were the most common other parameter classes (22 and 15 articles). For the treatment type, 36 articles dealt with *engineered treatment processes*, with the majority, 24 studies, focusing on wastewater treatment and water reuse. The various steps of drinking water treatment, as well as stormwater and greywater treatment, were the topics of the other 12 articles. The *attenuation of microorganisms during groundwater transport* was the focus of eight studies. In total, five of these involved natural tracers, and three involved injected tracers. Riverbank filtration, managed aquifer recharge and the drinking water treatment step of slow sand filtration were found to be the main processes studied. Investigations of microorganism attenuation express changes in treatment indicator concentration during a treatment step as percentage reduction or as log_10_ reduction values (LRV, the difference in log_10_-transformed concentrations before and after the treatment step; Momba et al. 2019).

In summary, the identified studies using GFPD, as representatively shown below, predominantly focus on nucleic acid target concentration changes, as an indication for the decrease of cell and virus concentrations during biological wastewater treatment or aquifer transport. Importantly, investigation of water treatment processes often also determine disinfection efficacies by characterizing the microbicidal and virucidal effects on FIO and pathogens (section ‘*Generating viability – and infectious status information by molecular tools*’ in the section ‘*Discussion*’). Viability PCR and enzymatic treatment PCR (ET-qPCR) are molecular techniques used to assess the viability and infectious status of microorganisms. In our systematic search, three articles were identified that employed these methods.

#### Evaluating microorganism removal during engineered treatment processes

##### Detection of nucleic acids

For the characterization of the removal of pathogens, such as viruses, through wastewater treatment, viral qPCR MST markers have been increasingly used and offer some advantages over traditional indicator viruses such as phages. The most important aspect of qPCR MST markers is that their concentrations in untreated wastewater are expected to be far greater than those of most viral pathogens (Hughes et al. 2017, Kitajima et al. 2018). This is particularly important because an indicator whose concentration is high can be detected consistently and more easily in different stages of treatment processes. The concentrations of coliphages in wastewater were found to be 7-log_10_ PFU/l, while the concentrations of enteric viruses such as human adenovirus and human polyomaviruses were variable and reported to be on the scale of 6 to 9-log_10_ copies/l (reviewed in Ahmed et al. 2020). Several studies have reported high numbers of PMMoV, crAssphage, *Bacteroides* (HF183) and *Lachnospiraceae* (Lachno3) and other qPCR MST markers in untreated wastewater (Rosario et al. 2009, Hughes et al. 2017, Ahmed et al. 2018, 2019). Furthermore, qPCR MST markers show little variation in untreated wastewater, and the concentrations range between 8 and 10 log_10_ copies/l (Hughes et al. 2017, Ahmed et al. 2019).

Several studies determined the log reduction values of human MST qPCR markers such as crAssphage and PMMoV in full-scale WWTPs (reviewed in Ahmed et al. 2020, Sabar et al. 2022). For example, Hamza et al. (2011) reported an ~3-log_10_ reduction in PMMoV concentrations in a conventional activated sludge treatment plant in Germany, which was similar to the reduction in polyomavirus and torque teno virus. Hughes et al. (2017) reported an ~1.1-log_10_ reduction in PMMoV concentrations in an activated sludge WWTP, which was less than those of HAdV and HPyV but similar to those of norovirus and enterovirus. Similar log reduction value of PMMoV was reported by Kuroda et al. (2015) in a WWTP in Vietnam. Schmitz et al. (2016) reported < 1-log_10_ reduction of PMMoV during activated sludge and biological trickling filter and the reduction rate was similar to aichivirus, norovirus, sapovirus, adenovirus, and polyomavirus. Based on the log reduction values reported in the literature PMMoV appears to be a conservative viral indicator for the reduction of pathogenic viruses in WWTPs. Several studies reported the reduction of crAssphage ‘the most abundant [known] virus’ in the human gut in WWTPs with activated sludge. Tandukar et al. (2020) reported a log reduction of 3.3 log_10_, while (Farkas et al. 2018) reported 1.0–2.0 log_10_ reduction.

Asami et al. (2016) determined the log_10_ reduction of PMMoV and JC polyomavirus for coagulation-sedimentation and rapid sand filtration processes in a drinking water treatment plant (DWTP) in Bangkok, Thailand using qPCR. The observed removal efficiencies varied depending on treatment step, season, and raw water quality, with LRVs ranging between 0.4 and 1.6 for PMMoV and between 0.5 and 1.9 for JC polyomavirus.

##### Molecular strategies to indicate the viability and the infectious status

The original idea of applying viability qPCR to bacterial MST markers was to gain information on recent faecal pollution events in water resources (Bae and Wuertz 2012, 2015). More recently, Jager et al. (2018) used qPCR with and without propidium monoazide (PMA) pretreatment as well as cultivation-based methods for *E. coli*, enterococci and *P. aeruginosa* to evaluate the removal efficiency of wastewater ozonation, a tertiary treatment step. PMA is an intercalating DNA dye that penetrates cells with impaired membranes and prevents PCR-based amplification (Nocker et al. 2006). It thus allows for selective detection of viable cells. PMA-qPCR is, therefore, also known as viability qPCR. FIO removal rate estimates were ranked in the following order: cultivation-based > viability qPCR > qPCR (Jager et al. 2018), emphasizing the differences among the culturable population, the viable but not culturable population and the total bacterial DNA. Viability qPCR, in comparison with qPCR, has also been applied to FIO (*E. coli*) and bacterial and viral MST markers (crAssphage, JC, and BK polyomavirus, human adenovirus, human-associated *Bacteroides* HF183) in sewage sludge flocs to assess their removal and inactivation during potassium ferrate treatment (Wang et al. 2023). Spatial distribution and movement resulting from the potassium ferrate treatment of the FIO and MST markers could be analysed in different compartments of the sludge flocs, encompassing various extracellular polymeric substance fractions. The reduction of the MST marker determined by qPCR was up to two orders of magnitude lower than the reduction determined by viability qPCR (Wang et al. 2023).

Similarly, enzymatic treatment qPCR (ET-qPCR), which applies enzymatic treatment using proteinase K and RNase, was used to estimate infectivity of bacteriophage MS2 in water (Pecson et al. 2009). By utilizing multiple-PCR-amplicons (providing whole genome coverage) and partial inactivation using different virucidal agents (such as heat, UV-B light, and singlet oxygen), the authors demonstrated that genome damage does not fully explain viral inactivation. Therefore, PCR-based assays would never yield results equivalent to infectivity assays. These assays fail to completely account for specific false positives that may arise when testing for MS2 bacteriophages. Consequently, to effectively monitor MS2 infectivity using ET-qPCR, it becomes crucial to determine a statistical ratio of total inactivation by cell culture in advance. Therefore, this calculation should be established beforehand for the applied treatment conditions and the given virus, but culture methods are not available for all human pathogenic viruses (Pecson et al. 2009). A follow-up study investigating UV-C treatment and relying on qPCR without pretreatment demonstrated that viral inactivation may be estimated in conjunction with mathematical models for JC polyomavirus and HAdV (Calgua et al. 2014). For more discussion on this topic, please refer to the section *'Direct detection of nucleic acids: characteristics and challenges'* in the *'Discussion'*.

#### Evaluating microorganism attenuation in groundwater

Pathogen removal during subsurface passage may be studied by investigating infiltrated faecal pollution (e.g. managed aquifer recharge), and monitoring the removal of pathogenic or indicator microorganisms. One way to investigate pathogen removal is to analyse water samples for naturally present microorganisms along a transect. Another way is with tracer tests using an injected target microorganism or surrogates. This can be done either as a laboratory experiment, using columns packed with aquifer material, or in the field.

The vast majority of such transport studies quantify microbial targets with microscopy or cultivation-based methods. Using genetic tools to quantify surrogate or pathogenic organisms (i.e. bacteriophages and enteric viruses) for groundwater transport studies is a relatively novel application of this technology, and therefore, limited literature exists. These approaches allow innovative analyses such as the quantification of multiple microorganism cotransport using multiplex qPCR and differentiating between infectious and inactivated viruses, when qPCR is used together with culture techniques (Betancourt et al. 2014, Bellou et al. 2015, Wang et al. 2022). In addition, genetic methods are a reliable way to enumerate microorganisms attached to particles, such as sediment and microplastics (Hassard et al. 2016). Genetic tools can also be used to confirm possible false-negatives derived from microscopy or cultivation-based methods. This is especially useful, as field tests are often expensive and labour intensive, and practical (small) sampling volumes often yield negative results. Low concentrations of target organisms require sampling larger volumes, which often presents additional challenges (Haramoto et al. 2018, Forés et al. 2022).

##### Natural tracers

Managed aquifer recharge involves natural subsurface processes to treat intentionally infiltrated surface water or wastewater effluent. In a study of the treatment efficiencies of three such systems in the USA, (Betancourt et al. 2014) measured viral pathogens and PMMoV, a human-associated viral marker, by qPCR in the infiltrated water and in a series of wells, providing the log reduction rates over given distances. Near the highly polluted Rocha River in Bolivia, surface water and riverbank filtrate are often used for irrigation, another example of indirect wastewater reuse (Verbyla et al. 2016). The removal (log reduction) during riverbank filtration was assessed for this study using reference pathogens recommended for wastewater reuse, PMMoV, as well as a human-associated bacterial indicator, and a QMRA of the consumption of the irrigated lettuce was performed.

##### Injected tracers

If the aim is to study the transport of pathogenic microorganisms in field tests, a surrogate is often used as a tracer, that mimics the pathogen in size and surface characteristics, while die-off rates are determined separately using batch tests. The transport of the surrogate can be compared to the pathogenic microorganism in small column tests in the laboratory, using aquifer material, while the surrogate is injected or applied at a field site. In this way, it is possible to upscale the transport of dangerous substances using transport models. With this goal in mind, Stevenson et al. (2015) used qPCR to quantify the transport and removal of HAdV and its surrogate, PRD1 phages, in small column tests. In regards to water treatment, the removal of *Cryptosporidium parvum* and its surrogate *Clostridium perfringens* by slow sand filtration was evaluated by Hijnen et al. (2007) as the last step in drinking water treatment using water taken from the Rhine River and spiked with the microorganisms. *C. perfringens* was enumerated using cultivation, and the colonies identified with PCR. Bauer et al. (2011) used qPCR to analyse enteric adenoviruses to evaluate the efficiency of slow sand filtration and river bank filtration as drinking water treatment steps. Wang et al. (2022) investigated the transport of MS2 phages, a surrogate for enteric viruses, from a surface water pond to groundwater via riverbank filtration. The authors differentiated between infectious phages by plaque assay versus the total number of phages detected by qPCR.

##### Synthetic tracers

A unique application of genetic tools is using synthetic DNA as a tracer which can be employed as multipoint tracers thanks to the practically unlimited sequence options and their specific quantification using qPCR (Dahlke et al. 2015, Pang et al. 2022). Another innovative idea is the use of DNA-labelled microspheres as surrogates for pathogenic microorganisms (Pang et al. 2014). This enables the enumeration of the pathogen and its surrogate by the same analytical procedure, qPCR, allowing more direct comparability.

### Application 5: infection and health risk assessment

GFPD are increasingly applied to support infection- and health risk estimation regarding human usage of water and water resources. The range of applications is very broad and includes guidance in hazard identification (e.g. reference pathogen selection), calibration of fate, transport, and QMRA models targeted to specific sources, and the genetic detection of risk indicators and markers, as alternative to cultivation-based enumeration techniques.

The study design analysis found 26 articles that estimated health risk by the support of GFPD: seven epidemiology studies at ‘*recreational*’ water sites and 19 QMRA studies, most of which were conducted in ‘*recreational*’ waters, five focussed on ‘*drinking*’ water and one on ‘*irrigation*’ water. The epidemiology studies compare ‘*traditional general faecal markers*’ with illness rate, while the QMRA studies apply ‘*MST markers*’ to QMRA, using one of the above approaches. The most prominent parameter classes are ‘*traditional general faecal markers*’ and ‘*MST markers*’ (*n* = 7 and *n* = 21, respectively), measured by qPCR. The relevance of obtaining information on the viability- or infectious status for infection and health risk assessment is addressed in the ‘*Discussion*’ section (‘*Direct detection of nucleic acids: characteristics and challenges*’).

#### Guidance in hazard identification for QMRA

Host-associated faecal marker quantification in water resources can guide reference pathogen selection for QMRA. This concept has been included in the framework of integrated faecal pollution analysis and management (‘3-step approach’) of karstic drinking water resources (Farnleitner et al. 2018, Savio et al. 2018). The three steps involve (1) catchment pollution source profiling, (2) monitoring of general faecal pollution, and finally, (3) hypothesis-guided qPCR MST marker enumeration in spring water. At a large, complex and hardly accessible alpine karstic spring water catchment with importance for public water supply in Austria, the results pointed at zoonotic pathogens from ruminants, including cattle as the priority QMRA reference targets (Reischer et al. 2011, Savio et al. 2018). The approach introduced by Farnleitner et al. (2018) was later extended to urban river catchments using probabilistic modelling to simulate the occurrence and extent of faecal pollution sources in parallel with zoonotic pathogens from direct human as well as indirect livestock and wildlife faecal pollution sources (Derx et al. 2023). The probabilistic estimates from the catchments and the direct measurements in the river indicated that combined sewer overflows and communal WWTPs were the largest contributors to faecal pollution at the studied site. The developed approach was indicated to be a robust basis for microbial fate and transport modelling and for QMRA (Derx et al. 2023).

MST qPCR marker analysis was also used to associate cases of human illness predicted by QMRA with bovine, human, or unknown sources in contaminated private wells in Wisconsin, USA. Although some of the cases of illness were indicated to be of human pollution origin, the results suggested that most of the cases were caused by bovine faecal pollution. This outcome had important implications for land use and water safety and health risk management of the fractured aquifers (Burch et al. 2021). In a study in the Netherlands, MST qPCR marker analysis was applied to trace back the origin of infection risks from *Campylobacter sp*. at a stormwater collection site (water plaza). The presence of human MST markers indicated a cross-connection with the combined sewer system (Sales-Ortells and Medema 2015).

Importantly, the performance characteristics (e.g. faecal sensitivity and specificity) of MST markers as well as their application design have to match the infection and health risk characteristics of the human and zoonotic pathogens considered (e.g. specific infectivity, specific health burden) to avoid masking of faecal hazards and their associated risk levels (Table 2).

**Table 2.** Overview of essential biological–diagnostic attributes of faecal indicators and associated genetic targets. The overview shows the ‘big five’: sensitivity, specificity, persistence, resistance, and mobility. Most of the shown attributes can be divided into subcharacteristics. Various methods and specification metrics have been suggested for determination.

Characteristic	Basic definition	Remarks	Methods/metrics
**Faecal sensitivity**	Occurrence of faecal indicator or genetic target in faecal pollution source(s) to be indicated.	Should be ubiquitous and abundant in targeted pollution source(s). Indicator/genetic target of **total faecal pollution** in human and vertebrate animal faecal pollution sources (primary enteric habitats). **MST** indicator/genetic marker only in faecal pollution-source-groups to be indicated (human, ruminant, pig, and so on).	**Incidence** or (**binary**) **faecal sensitivity** (% presence in targeted source; e.g. Ahmed et al. 2009, Farnleitner et al. 2010, Shanks et al. 2010a, b); **Abundance** in target excreta or wastewater (conc. per volume/mass; Farnleitner et al. 2010, Ervin et al. 2013, Mayer et al. 2018)
**Faecal specificity**	Nonoccurrence of faecal indicator or genetic target in the pristine environment or nontargeted compartment	Indicators/targets of **total faecal pollution** should be absent in pristine environments not polluted with faeces. **MST** indicator/marker also absent in nontargeted faecal pollution sources (e.g. human faecal marker not in ruminant excreta).	**False-positive occurrence** in pristine habitats (concentration per volume or mass; Vierheilig et al. 2012) (**Binary**) **faecal specificity** (% presence in nontargeted source groups; e.g. Shanks et al. 2010a,b, Linke et al. 2021) **25th/75th percentile discrimination metric** (25th percentile of target minus 75th percentile of nontarget, log-transformed concentrations (Reischer et al. 2013)
**Persistence**	Extent of survival (i.e. viability) of indicator or molecular detectability (i.e. nondegraded amenable nucleic acids) of genetic target in the (aquatic) environment.	Persistence varies widely among microorganisms and genetic targets and is influenced by many potential abiotic and biotic ecological factors, such as sunlight, temperature, salinity, grazing, and so on.	**T90, T99** [time in days needed for a 1 log_10_ (T90) or 2 log_10_ (T99) reduction in indicator or genetic target concentration (Mitchell and Akram 2017)] **Decay rate coefficient *k*** of, e.g. a first-order decay model (Chick 1908, Balleste and Blanch 2010)
**Resistance**	Extent of survival (i.e. viability, proliferation, and infectivity) or molecular detectability (i.e. nondegraded amenable nucleic acids) of indicator or genetic target, respectively, towards chemical substances (e.g. metals, antibiotics) and during technical treatment and disinfection processes.	Resistance varies widely among microorganisms and genetic targets and is influenced by many chemical and physical factors, such as type of chemicals (chlorine, ozone,), concentration and contact time (ct-value), temperature and time in thermal processes, and fluence in UV irradiation.	**Inactivation rate and kinetics** obtained under carefully controlled conditions (Hoff and Akin 1986) Inactivation rate constants **Log reduction** of the concentration of microorganisms/pathogens; a measure for the effect of a substance or for the efficacy of the process (Guerrero-Latorre et al. 2016). The log reduction to be achieved for a target is determined by risk assessment.
**Mobility**	Transport characteristics of the indicator or genetic target in the (aquatic) environment	Mobility is influenced by many factors, such as mass and size of the microorganism/phage, its attachment and aggregation behaviour (electrostatic and hydrophobic forces), its detachment behaviour, as well as the motility of certain microorganisms. Mobility characteristics may change as the microorganism decays.	**Sedimentation** onto the river bed applies to larger-sized microorganisms (protozoa) or microorganisms attached to sediment (Jiang et al. 2015, Wu et al. 2019) **Resuspension** of microorganisms attached to the riverbed sediments (Jamieson et al. 2005, Kim et al. 2010, Park et al. 2017) **Straining** due to microorganism size and aquifer material grain size distribution e.g. (Bradford et al. 2003, Tufenkji et al. 2004) **Attachment/detachment** (Schijven and Hassanizadeh 2000) **Motility** (Becker et al. 2004)

#### Calibration of catchment models to estimate pathogen concentrations for QMRA

Genetic faecal marker quantification also proved valuable for catchment-based QMRA modelling of faecal pollution sources. One of the principles of the ‘QMRAcatch’ philosophy is the catchment-specific calibration of microbial transport (i.e. dilution, advection, and dispersion) and fate (i.e. decay/persistence) models for specific faecal pollution sources by the use of MST markers. The calibrated and verified models can be used to derive pollution and management scenarios for given points of interest (e.g. drinking water abstraction sites) based on pathogen transport/fate simulations. Reference pathogens are quantified in pollution sources or derived from epidemiological data and the literature (Schijven et al. 2015, Derx et al. 2016).

In a scenario analysis considering river water as a raw water source for drinking water production, the authors calibrated QMRAcatch for human faecal pollution pathways, such as from communal wastewater disposal, using human-associated MST qPCR marker data for the Austrian section of the Danube River (Demeter et al. 2021). By use of a conceptual semidistributed hydrological model and regional climate model outputs, the authors simulated the interplay of future changes (e.g. climate change, population) and wastewater management measures (enhanced WWTP treatment, prevention of combined sewer overflows) with respect to the infection risks for viral and bacterial reference pathogens (Demeter et al. 2021). The study demonstrated that the degree to which future changes affect drinking water safety strongly depends on the type and magnitude of faecal pollution sources, and is thus highly site- and scenario-specific.

More recently, the modelling approach was extended towards source-specific calibration to multiple faecal pollution sources, using MST markers for humans, ruminants, pigs, and birds. An improved hydrological module (2D hydrodynamic flow, rainfall-runoff, and differential MST decay) allowed comparing external (allochthonous) and internal (autochthonous) faecal pollution sources and their associated infection risks from zoonotic parasites (*Giardia, Cryptosporidium*) for the Danube River (human wastewater input) and its floodplains (animal sources) downstream of Vienna (Derx et al. 2021). An important result for best management practices is that autochthonous and allochthonous faecal sources during flood and rainfall events contributed pathogen loads with similar orders of magnitude.

#### Infection and health risk indicator role trough epidemiological studies

The traditional method of recreational water quality monitoring of surface waters has been based on the application of cultivation-based FIOs. For example, the relative risk of illness for swimmers and nonswimmers in recreational waters was estimated based on cultivation-based enterococci levels (USEPA 1986). However, a revision of these guidelines in 2012 (‘NEEAR study’) reported that qPCR measurements of general enterococci concentrations are better predictors of the rate of gastrointestinal illness among swimmers in recreational waters compared to cultivation-based enterococci levels (USEPA 2012a). This study established a combined approach, using cultivation-based *E. coli* enumeration (beach action value of 235 CFU per 100 ml of water) and genetic enterococci qPCR quantification (beach action value of 1000 calibrator cell equivalents per 100 ml) with a health-based compliance target of 36 cases of gastrointestinal illnesses per 1000 swimmers (USEPA 2012b).

MST marker quantification by qPCR has also been incorporated in epidemiological studies. For example, Griffith et al. (2016) applied several bacterial and viral indicators to predict gastrointestinal illness in three Californian beaches (*n* = 10 785 swimmers) by comparing qPCR and cultivation-based methods. At one beach, human-associated genetic MST marker levels displayed the highest associations with gastrointestinal illness. The authors concluded that performance of a selected parameter is likely site-specific. Napier et al. (2017) conducted a prospective cohort study also using human-associated genetic MST markers in water (self-reported gastrointestinal illness among 12 060 swimmers at six beaches across USA). Inconsistent associations were noted between results; however, the authors concluded that qPCR MST marker data may be useful in assessing human health risks in recreational water bodies.

#### Infection and health risk indicator role through indicator to pathogen ratio and QMRA

An increasing number of studies have attempted to establish a link between genetic MST marker concentrations and infection risks in recreational waters using a QMRA modelling framework. One of the first studies of this type was conducted to estimate the risk of gastrointestinal illness for adults swimming in waters contaminated with untreated sewage (Staley et al. 2012). In this study, norovirus was selected as the reference pathogen. The HF183 marker was detected in sewage dilutions indicating gastrointestinal illness risks greater than or equal to the benchmark value of 10/1000 primary contact recreators in several sampling sites based on the 1986 Ambient Water Quality Criteria (USEPA, 1986). Boehm et al. (2015) established a relationship between concentrations of the human-associated qPCR markers HF183 and HumM2 and gastrointestinal illness risk of swimmers in recreational waters using a QMRA approach. The authors noted that the benchmark gastrointestinal illness rate of 30/1000 primary contact recreators occurred when the median concentrations of HF183 and HumM2 marker genes were 4200 and 2800 GC/100 ml of water, respectively. In a subsequent study, Boehm et al. (2018) incorporated the decay of both human faecal-associated markers and norovirus in the model to determine the risk associated with scenarios in which the age of contamination is unknown or water is contaminated by fresh untreated sewage. When an untreated sewage contamination scenario was considered, the risk-based threshold was ∼9700 GC/100 ml. The analysis suggested that a risk-based threshold of 4100 GC/100 ml is warranted for the HF183 marker gene when the age of contamination is unknown. Schoen et al. (2020) modelled risk-based thresholds across different mixture and sewage-age scenarios for crAssphage, HF183 and polyomavirus using QMRA. The authors concluded that genetic markers may not be effective when aged sewage contributes most pathogens relative to fresh contamination. Similar risk-based MST marker thresholds have also been estimated for gull *Catellicoccus*, human *Bacteroides*, and human *Lachnospiraceae* markers (Brown et al. 2017, Boehm et al. 2018, McLellan et al. 2018).

Such information can be extremely valuable to regulators in interpreting quantitative MST marker data concerning potential human health risk and developing plans for faecal pollution mitigation and to assess human health risks more accurately (Zhang et al. 2019).

### Application 6: outbreak tracing and wastewater surveillance

The GFPD toolbox has also proved useful in fields that traditionally focus on the detection and characterization of pathogens, such as waterborne disease outbreaks or pathogen transmission route characterization. Twenty outbreak and pathogen transmission tracing articles were retrieved, predominantly employing ‘*MST markers*’ with paired measurements of ‘*pathogens*’ and ‘*cultivation-based FIO*’. Additionally, five of the retrieved articles applied MST markers in wastewater surveillance for SARS-CoV-2. Given the importance of this topic, additional literature searches were performed and revealed three different roles in which MST markers may be implemented for wastewater surveillance.

#### Outbreak tracing, disease transmission routes, and sanitation trials

Waterborne disease outbreaks occur worldwide and may be caused by several factors, e.g. in the case of drinking water, these may include raw water contamination, treatment deficiencies, and drinking water distribution network failures. Tracing an outbreak is done predominantly by tracking the pathogen strain from patients through the transmission routes back to the exposure source by genetic typing and sequencing (molecular epidemiology, e.g. Popa et al. 2021). Alternatively, host-associated genetic faecal indicators can help identify the source for contamination and support the elucidation of disease or pathogen transmission routes. While they provide less specific outbreak-related information compared to pathogen typing, these markers are much more abundant than the pathogen in question, making them easier to detect in the environment. For example, host-associated markers were used in outbreak studies in Finland with ~450 illness cases to identify the source of pollution and to ensure the success of contaminant removal from the drinking water distribution system (Kauppinen et al. 2019). A novel approach used the human-associated genetic marker HF183 in a norovirus outbreak involving 179 cases in Pennsylvania, USA. It was applied as a microbial tracer to demonstrate the hydrogeological connection between a malfunctioning septic system, drinking water well, and recreational water area and, therefore, helped inform outbreak prevention strategies in the area (Mattioli et al. 2021). The coastal Biobío Region of Chile had been affected by repeated hepatitis A outbreaks. Human mitochondrial DNA, faecal coliforms, and live microbial biomass correlation was investigated and the concordance between human faecal pollution in the coastal waters and a seasonal hepatitis A outbreak strongly suggests that the investigated parameters can be used as a proxy to evaluate the risk of outbreaks of thalassogenic diseases (González-Saldía et al. 2019). During a large *Campylobacter* outbreak in Norway with over 2000 cases and 76 hospitalizations, an old cave used as a drinking water pool was identified to be faecally contaminated as indicated by the presence of *E. coli*. Host-associated genetic markers for humans, ruminants, horses, pigs, and other animals were applied to generate a faecal source distribution profile. This revealed that the faecal contamination was likely zoogenic in origin (horses) (Paruch et al. 2020).

In settings with poor sanitation facilities and practices, pathogen transmission routes can be multiple, therefore, planning WASH interventions to reduce pathogen exposure is challenging. A study in an urban slum in Nairobi, Kenya, set out to separate two types of human faecal waste, originating from children and from adults, because mitigation steps to reduce contamination could differ (Bauza et al. 2019). Using 16S AmpSeq analysis of faeces from both cohorts and various surfaces and waters, as well as the algorithm SourceTracker, the authors identified child faeces as the dominant pollution source inside households, whereas faecal pollution from adults was more prevalent outside households.

GFPD tools can also be used to evaluate WASH interventions. A controlled, before-and-after trial was performed in neighbourhoods of Maputo, Mozambique to estimate the potential health impacts of a sanitation intervention (installation of improved pit latrines). The authors first assessed the transmission routes through a comprehensive sanitary, environmental, and socioeconomic survey, including the measurement of a set of general and host-associated faecal indicators. They found widespread faecal contamination in soil, water, and food preparation surfaces, including from human sources. However, faecal contamination levels were largely disconnected from these analysed factors (Holcomb et al. 2020). In the before-and-after trial, the authors used a Bayesian hierarchical modelling approach to account for MST marker performance. Bootstrap estimates found no effect of the sanitation intervention on the prevalence of general and human-associated indicators, which highlights the complexity of the system and the need for multisectorial, ‘transformative’ WASH interventions (Holcomb et al. 2021).

#### Wastewater surveillance

Wastewater surveillance, also called wastewater-based epidemiology, seeks to relate the occurrence of a public health target of interest measured in wastewater to the public health of a respective population (e.g. Choi et al. 2018, Lorenzo and Picó 2019). COVID-19 gave a strong boost to the field, where SARS-CoV-2 RNA occurrence in wastewater is used as a proxy for the prevalence and dynamics of the infection in the population (Ahmed et al. 2022). In contrast to HRWM, which focuses on the users of the water (e.g. drinking water, recreation, and irrigation), wastewater surveillance is an ‘upstream approach’, looking back at the population’s health. Samples for wastewater surveillance are taken from raw wastewater collected by centralized sewer systems. Surface waters heavily contaminated by sewage may also exhibit an epidemiological indicator function in terms of wastewater surveillance (e.g. (Kolarević et al. 2022, Maidana-Kulesza et al. 2022).

Successful wastewater surveillance applications require the accurate measurement of public health targets in wastewater. However, this can be challenging because the proportion of human waste in a wastewater sample can be highly variable in time and space (i.e. between/within sampling site variability). In addition, the sample matrix may be challenging from an analytical point of view. In response, many scientists have suggested to use faecal markers (e.g. PMMoV, crAssphage, HF183) to support sample characterization and provide quality control in wastewater surveillance.

One application category is the characterization of surveillance samples, which mainly aims to quantify the human faecal levels in (waste)water but could also be used to characterize other animal sources. One study examining the epidemiological indicator function of SARS-CoV-2 in surface waters for countries with poor wastewater treatment, e.g. applied an advanced sampling site characterization approach including measurement of human- (BacHum), ruminant- (BacR), and pig- (Pig-2-Bac) associated genetic faecal markers. By using this approach, they could trace and identify sites with significant raw sewage influence from human populations, which may serve as sampling locations for wastewater surveillance where no obvious sewage outlets exist (Kolarević et al. 2022).

In addition, MST methods have also been used as internal process controls within wastewater surveillance investigations, either as a proxy for the public health target of interest to ensure adequate recovery and/or as performance metrics of sampling/sample processing protocols. In a monitoring study of SARS-CoV-2 in the wastewater and rivers of Tapachula (southern Mexico), e.g. PMMoV was not only used as a faecal pollution marker but also as an analytical control to confirm RNA extraction and amplification (Zarza et al. 2022). In another study investigating the intraday variability in 1-h and 24-h composite wastewater samples, the concentrations of the human viral indicators crAssphage and PMMoV were monitored in addition to the less prevalent human pathogen adenovirus (HAdV) to inform the design of appropriate wastewater sampling strategies for wastewater surveillance (Ahmed et al. 2021).

The most widely observed use of faecal markers for wastewater surveillance was the normalization of pathogen occurrence data. In this context, different MST markers were used either to describe spatial and temporal trends of the public health target of interest or to support the prediction of community infection trends. For example, Wolfe et al. (2021) describe how normalizing SARS-CoV-2 concentrations from multiple WWTPs with PMMoV can be used to compare the incidence of laboratory-confirmed new COVID-19 cases by accounting for variability in recovery and differences in human faecal loads within or between WWTPs. Another study investigated the suitability and performance of various normalization parameters and how well they correlated with local clinical cases. Normalization by crAssphage and PMMoV (amongst others) was found to show varying performance for different sampling sites (Mitranescu et al. 2022). Similar findings were described for PMMoV in a study by Nagarkar et al. (2022) suggesting that the most suitable faecal marker for normalization may vary by site and wastewater management practices.

Wastewater surveillance represents an exciting new application for GFPD. However, additional research is warranted, especially in areas highly relevant for wastewater surveillance, such as the behaviour of MST targets in sewer systems, distribution between hosts, or protocol performance assessments with wastewater sample processing methods. Although genetic faecal markers have already proven to be valuable, it remains unclear which of the many available methods are most suitable. Optimal method selection will likely vary by use scenario, surveillance target, and geographic location. In addition, applications will likely not be restricted to MST markers, but will use the entire methodological capacity of GFPD.

### Application 7: other applications

Assessing water resources for the possible presence of faecal pathogens is the foundation of GFPD. However, these tools have also proven useful in other arenas. For example, 48 out of the 107 articles in this category had antibiotic resistance as the primary research focus, complemented with a GFPD method, mostly MST pertaining to markers. In total, 12 articles used MST markers to trace nutrient inputs into ambient waters. Interestingly, three articles were observed from the archaeology field, and employed genetic methods for faecal bacteria. These three disciplines are further discussed below.

#### Identification of the sources of ARGs

Antimicrobial resistance (AMR) is one of the top 10 global public health threats (World Health Organisation 2021). The spread of antibiotic resistant bacteria (ARB) and their ARG from hotspots such as WWTPs or agricultural run-off into freshwater and coastal ecosystems is of growing concern (Gao et al. 2018). Identifying such hotspots is, therefore, a pressing issue. Beyond the monitoring of a large panel of ARB and ARG targets of concern and the genotyping of ARGs (similar to pathogen typing), two additional approaches have been established that allow tracking their source.

The first relies on the differing AMR patterns of the gut microbiota of various host species, reflecting the differing antibiotic usage in human and veterinary medicine. This differing pattern is exploited for MST, where the pattern of the environmental samples of unknown pollution profile is compared to a library of known faecal sources. In the early 2000s, this ‘antibiotic resistance analysis’ relied on the phenotypic AMR characterization of *E. coli* or enterococci isolates (see also the section ‘*The early days of genetic methods for faecal pollution diagnostics*’; Mott and Smith 2011). More recently, Li et al. (2018) adapted the Bayesian source tracking tool SourceTracker, originally relying on 16S AmpSeq data, to ARG data from whole metagenome sequencing. At two rivers in China with dense human and livestock populations and with excess nutrient levels, this tool identified WWTPs as the major source of ARG at the majority of sites (Hu et al. 2020). At one site, nonhuman animal faeces proved to be the major pollutant. Correlations with host-associated faecal indicator genera, identified based on 16S AmpSeq data, helped identify swine manure as the main nonhuman faecal input.

The second approach relies on the co-occurrence of host-associated faecal microorganisms and ARG and/or ARB, because of a common source. Williams et al. (2022) studied persistent faecal pollution in an urban coastal bay in Sydney, Australia. qPCR MST and 16S AmpSeq together with SourceTracker were employed to pinpoint which stormwater drains drive dry-weather or wet-weather faecal pollution. Significant correlations between ARGs and the human-associated MST marker HF183 showed that the same stormwater drains were the main sources of ARG and of human faecal pollution. The Bolivian Andes is an intense mining area, and heavy metals exert selective pressure for the coselection of ARGs. Through multiple linear regression between the first principal component of a PCA of ARG data as dependent variable and metals, the human-associated viral marker crAssphage and physicochemical parameters as independent variables, Agramont et al. (2020) demonstrated that it is likely that human wastewater inputs, rather than heavy metals, drive ARG concentrations in the three rivers studied.

#### Identification of the sources of nutrient inputs

Nutrients, such as nitrite (NO_2_^−^), nitrate (NO_3_^−^), and phosphate (PO_4_^3−^), are essential for plant life. However, excess concentrations can lead to eutrophication and harmful algal blooms (Kendall et al. 2007, Fenech et al. 2012). In addition, ingestion of high amounts of nitrate, e.g. through drinking water, may have serious health consequences such as methemoglobinemia of infants (blue baby syndrome), colorectal cancer, and thyroid disease (Ward et al. 2018). The World Health Organisation Drinking Water Guidelines recommend setting thresholds of nitrate and nitrite concentrations in drinking water (WHO 2017) and some countries also regulate surface water and groundwater (European Union: 91/676/EEC and 2006/118/EC, within the frame of 2000/60/EC). Mitigation of excessive nutrient inputs is, therefore, a key water quality management task. Tracing nutrient inputs relies on the fact that ratios of rare to abundant isotopes of certain elements differ among environmental and biological compartments, due to isotopic fractionation during physiochemical and biochemical reactions. As a typical example, nitrate sources can be tracked using δ^15^N and δ^18^O isotopes (Kendall et al. 2007, Fenech et al. 2012). Since nitrate has numerous biotic and abiotic sources and isotope tracing cannot separate all source types, a toolbox approach is often useful, which can include MST markers (Fenech et al. 2012).

One of the early studies combining δ^15^N and δ^18^O isotope tracing with MST was conducted along the Sava River, a tributary of the Danube River, that crosses Slovenia, Croatia, Bosnia and Herzegovina, and Serbia. The combined results indicated that soil nitrification and human wastewater were the primary nitrate sources in the Sava River, and the latter was also the main faecal pollution source (Vrzel et al. 2016). Carrey et al. (2021) assessed the main sources of nitrate pollution in surface water and groundwater across Catalonia, Spain in a government-led effort to review vulnerable zones as defined by the European Union Nitrates Directive (91/676/EEC). Nearly 200 samples were analysed for multiple isotopes (δ^15^N, δ^18^O, δ^2^H, and δ^11^B from various molecules), viral and bacterial FIO, human-, ruminant-, and swine-associated MST markers and complemented by land use data. Each sampling location was interpreted individually. The conclusions from multi-isotopic and MST data agreed or partially agreed in 79% of the samples. The authors offered detailed discussion on the complementary nature of the two approaches and the possible sources of disagreement (Carrey et al. 2021). In the coastal areas of Southwest Florida, harmful algal blooms caused by elevated nutrient levels are a recurring problem. Malfunctioning septic tanks were suspected to be the source of nutrients. Brewton et al. (2022) applied δ^15^N and δ^13^C isotope tracing, elemental composition of particulate matter (C:N:P), a panel of nutrients, chemical tracers, cultivation-based FIO and human-, bird-, and gull-associated MST markers to tackle the complex challenge. These multiple lines of evidence pointed to a link between septic systems, groundwater, and surface water, ultimately resulting in harmful algal blooms. Additionally, chemical tracers and bird- and gull-associated MST markers indicated rainfall runoff to be a contributing factor (Brewton et al. 2022). The Changle River catchment in China has a high human population, intensive livestock farming (swine), and agricultural activities, all of which potentially contribute to the high nutrient levels of the river. A Bayesian isotopic mixing model using data from the nitrate dual stable isotope technique (δ^15^N- NO_3_^−^ and δ^18^O- NO_3_^−^) suggested manure and sewage to be the dominant pollution sources (Cao et al. 2022). Since nitrate isotopes cannot differentiate between manure and sewage, Cao et al. (2022) applied MST using 16S AmpSeq together with the algorithm SourceTracker, which suggested untreated and treated domestic wastewater as the main sources. Redundancy analysis brought all lines of evidence (isotopes, MST, land use, and various ions) together to reveal domestic wastewater as a probable cause of nutrient pollution (Cao et al. 2022).

#### Archaeology

Genetic markers can remain detectable much longer in sediments than in the overlaying water column (Korajkic et al. 2019). Sediments may therefore offer time-integrated information on faecal pollution. In a tidal freshwater marsh in South Carolina, USA, the ruminant-associated MST marker BoBac was found in all sections of a soil core, the deepest section of which dated to 1961 (Drexler et al. 2014). While in this hydrogeological system the bacterial community of fresh pollution might migrate through the layers, the findings provide evidence of at least recent, but potentially long-term faecal pollution likely from deer and/or cow manure. On much larger timescales, lake sediments may act as biological archives of sedimentary ancient DNA from autochthonous (in-lake) and allochthonous (from the catchment and beyond) sources (Capo et al. 2021). Among other tools, palaeoenvironmental enquiries into ancient human presence and pastoral activities may also use MST markers or DNA sequencing techniques (Capo et al. 2021). In a study in Northern France, the authors documented a shift from agro-pastoral practices to forested landscapes during the Roman period. Testing for ovine and bovine mtDNA markers revealed sheep as the dominant livestock before the transition (Etienne et al. 2015).

## Discussion

### Emergence of a new field in health-related water quality analysis

#### The advent of genetic faecal pollution diagnostics (GFPD)

Our search for peer-reviewed science regarding the analysis of faecal pollution-associated nucleic acid targets in water demonstrates the rapid development of genetic diagnostics within the field of HRWM since the start of the new millennium. The meta-analysis of the currently existing application types also highlights that this novel scientific discipline extends far beyond the enumeration of genetic MST markers. Many traditional HRWM aspects, such as treatment and microbial transport indications, infection risk assessment and QMRA, as well as integration into modelling and simulations were found to be supplemented by GFPD (sections ‘*Application 1*’ through ‘*Application 7*’). In addition, several novel aspects such as the support of epidemiological outbreak tracing, wastewater surveillance, and supplementing ABR research, have also been developed.

The emerging scientific field of GFPD still grows; no plateau phase is in view (Fig. 3). In the past decade, the focus of research has shifted from method establishment to the implementation of these methods in scientific field research. An emphasis on field implementation is also indicated by the frequent use of certain genetic faecal markers, with some of them already standardized at the national level (section ‘*Application 1*’). However, method development has not halted, and it is very likely that expected future technological developments in molecular biological analytics, sequencing and bioinformatics (e.g. Callaway 2022) will further promote diversification within the field of GFPD research.

It thus seems justified to define this emerging part of science as a new discipline: *genetic faecal pollution diagnostics* in health-related microbial water quality analysis (see the section ‘*Glossary*’). The aim of GFPD is to open up the ‘black box’ of microbial faecal pollution of water resources to support problem-oriented water safety management, covering aspects such as catchment protection and management, water quality monitoring, health risk management, and treatment requirement evaluation. Additionally, GFPD can be applied to areas outside the water sector, as exemplarily indicated by its use in archaeology (section ‘*Application 7*’).

#### GFPD analyses distinct nucleic acid-based faecal pollution signatures

Vertebrate gut microbial communities fundamentally differ from environmental ‘nondigestive’ microbial communities (e.g. water, sediment, soil, plant, and nonvertebrate), as first demonstrated by the meta-analysis of 16S AmpSeq data by Ley et al. (2008). Long coevolution between host vertebrate animals (including humans) and their intestinal microbiomes, driven by many selective forces (e.g. adaptive immune system, host selection pressure, and unique biochemical environment), is likely responsible for this clear distinction (Ley et al. 2008). Although cosmopolitan populations do occur, strong vertebrate gut-associations also exist on the individual taxa level of microorganisms (McLellan and Eren 2014, Youngblut et al. 2019, 2020, 2021). This clear intestinal versus nonintestinal microbial community dichotomy forms the essential basis of specific detection of faecal pollution in water, targeting nucleic acid-based signatures from gut-associated bacteria, archaea and viruses. Similarly, evolutionary adaptations between macro- and intestinal microorganisms also exist on the host level, providing the basis for MST (section ‘*Introduction*’).

**Figure 12.**
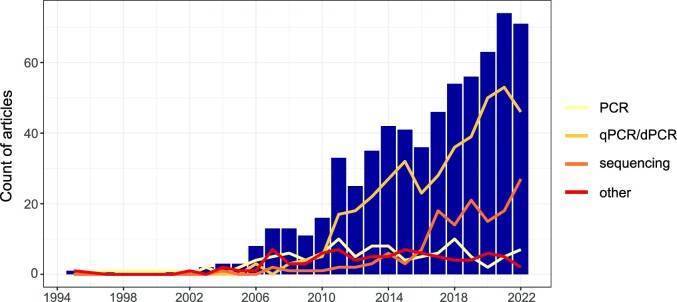
Analytical methods over the years in the ‘application studies’ pool, with the bar chart of all papers per year in the background (*n*_article_ = 649).

GFPD of today primarily focuses on the cultivation-independent detection of nucleic acid-based targets in the environment. The literature analysis highlighted that GFPD thus far predominantly relies on targeted analysis, where faecal pollution-associated sequences are directly detected by amplification methods (e.g. PCR, qPCR, and dPCR), using specific primers and probes. Owing to the enormous technological developments in HTS, nontargeted approaches, using broader taxonomic sequencing and subsequent specific *in silico* sequence alignment to faecal-associated signatures, have substantially improved during the past decade (Fig. 12, section ‘*Outcomes of the systematic study design analysis*’).

**Figure 13.**
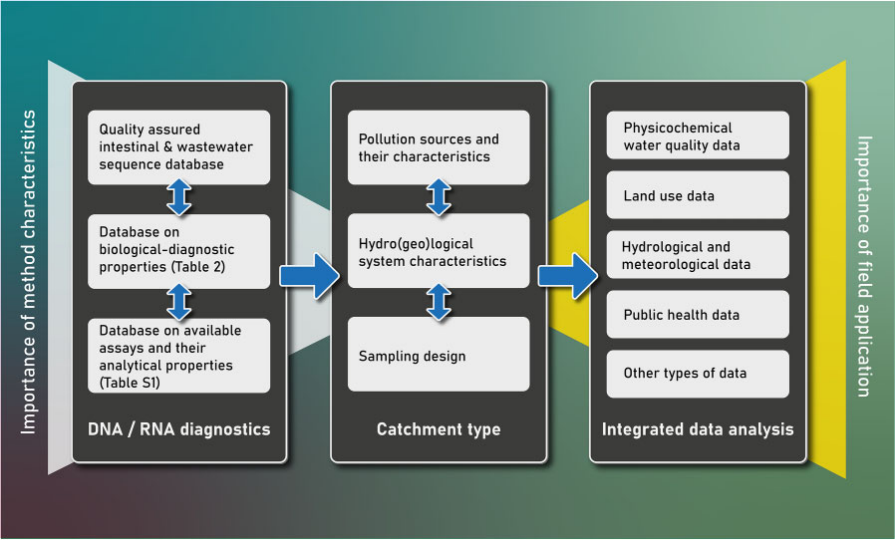
Conceptual framework of study design in the genetic faecal pollution diagnostics field.

Advances in intestinal microbiomics will certainly further benefit GFPD, expanding our understanding of ecophylogenetics and providing access to representative sequence databases to support, (i) *in silico* design and evaluation of molecular assays, and (ii) bioinformatic analysis of big data from HTS (Fig. 13). Human and other animal intestinal microbiome research, with the greatest relevance in life sciences and medicine, is a very young discipline, and much is expected to be achieved in the future.

### Identified revolutionary aspects of GFPD for HRWM research

#### Genetic faecal pollution detection and MST: a methodological quantum leap

The use of GFPD has fundamentally changed the way scientific questions on faecal pollution problems in the environment can be addressed and answered (Malakoff 2002). MST using genetic methods has opened the way to identify and quantify many different pollution sources that cultivation-based methods do not allow. Approximately, half of the identified GFPD studies (356 out of 649 articles) dealt with MST, i.e. the characterization and origin determination of faecal pollution. Many novel cutting edge GFPD studies, covering single and multiple sources in differing types of water resources, including elevated faecal pollution levels in watersheds, recreational waters, groundwater resources, aquaculture and others, could be successfully realized (sections ‘*Application 2*’ and ‘*Application 3*’).

#### Biobanking: a new key element in HRWM research

Traditional cultivation-based FIO analysis requires sample transport, processing, and subsequent cultivation within a short time period (usually <1 working day). This often significantly constrains the possibilities and extent of research. In contrast, GFPD enables long-term nucleic acid preservation (>1 year) before performing the diagnostic analysis (De Paoli 2005, Jackson et al. 2011, Cary and Fierer 2014).

The possibility of storing nucleic acids for posterior analysis has several essential implications for HRWM research. Assuming that there is sufficient capacity to establish a representative sample bank over time and space, researchers can (i) focus on selected samples of interest (e.g. pollution event-based analysis), (ii) focus on the parameters appearing most appropriate at the time of analysis, and (iii) extend the investigation to other samples and/or genetic parameters at any time, if sufficient analyte is available. In hydrological sciences, this type of sample archiving for posterior analysis (e.g. isotopes) has already been a standard practice for decades.

#### Nucleic acid sample transfer supports the globalization of HRWM research

Nucleic acid sample conservation during field work also opens the way to international network structures, useful for performing centralized analysis in specialized laboratories (Layton et al. 2013, Reischer et al. 2013, Mayer et al. 2018). This point is especially interesting for developing regions that lack the infrastructure for advanced GFPD. During the COVID-19 pandemic, infrastructures for molecular biological analysis were established in many urban centres throughout the globe and will likely contribute to centralized GFPD activities in the future. Thus, even advanced GFPD will not be limited to certain regions of the world but will be accessible from any remote location, provided that basic infrastructure for sample collection, processing, storage, and transfer, as well as standard operating procedures, are available.

### Direct detection of nucleic acids: characteristics and challenges

#### Characteristics of DNA/RNA-based target analysis

The literature analysis highlighted that GFPD targeting of prokaryotic microbiota (bacteria and archaea) has almost exclusively relied on DNA analysis, with the 16S rRNA gene as the most frequently used diagnostic region. In addition, alternative targets, such as gene regions for protein coding parts, have also been used (Shanks et al. 2008, Green et al. 2014a). The primary aim of tracing intestinal DNA signatures in the environment is the sensitive detection and characterization of faecal pollution. Such DNA analysis does not give any information about the physiological status of the targeted microbiota in the analysed water. Active, inactive, starving, viable but not culturable, or dead microbial populations are often detected equally. Depending on the applied extraction procedure, DNA attached to cells, organic debris, biofilms, or sediments, and even freely suspended DNA, is also detectable (Carini et al. 2016). The same is true for viral targets. Detecting viral DNA or RNA does not provide information on the infectious or noninfectious status of the targeted populations.

Notably, it was reported, that the application of ribosomal RNA via RT-qPCR for bacterial general faecal markers and MST markers increases the sensitivity and frequency of faecal pollution detection for several water resource types (Pitkänen et al. 2013). In addition, rRNA analysis may also be interesting for viability investigations (section ‘*Generating viability- and infectious status information by molecular tools*’).

#### Relevance of viability- or infectious status information

While the majority of genetic detection methods available do not account for information on the viability or infectivity status of the microorganisms or viruses from which the nucleic acids originate, it is important to note, that this is not the main purpose for many GFPD applications. For example, this is clearly the case for most of the identified faecal pollution detection and MST studies throughout the literature analysis (sections ‘*Application 1*’ to ‘*Application 3*’). Nevertheless, as outlined below, robust information on the persistence and resistance properties of the genetic targets is essential for the correct selection and application of genetic MST markers and for the appropriate data interpretation (section ‘*GFPD (MST) application frame: status quo and research needs*’). Other identified GFPD application areas, such as the support of outbreak tracing or wastewater surveillance, do not rely on the viability status of the microbial targets either (section ‘*Application 6*’).

Even the use in recreational water quality monitoring seems to be a realistic exercise, without the need for a viability endpoint (section ‘*Application 5*’). For example, a recent investigation on swimming-associated health risks, including 80 000 beachgoers at 13 beaches (pooled data), revealed the strongest associations between gastrointestinal symptoms and qPCR-quantified enterococci, but not with cultivation-based enumeration (Wade et al. 2022). It was previously hypothesized, that enterococci DNA, as quantifiable by qPCR, better reflects the survival of resistant pathogens during wastewater treatment (e.g. resistant enteric viruses) than cultivable enterococci concentrations (Wade et al. 2006, Srinivasan et al. 2011). Obviously, it is desirable for pathogen die-off kinetics to match the decay kinetics of the analysed indicator signals, irrespective of whether viability- or nonviability-based parameters are considered. Undoubtedly, more research is needed to better understand the principles behind these important relationships in GFPD and health risk assessment. However, the extent of already existing innovative research by nucleic acid-based qPCR analysis for infection- and health risk indication holds great promise for the future (section ‘*Application 5*’).

Information on viability or infectious status becomes an essential criterion when microbicidal and virucidal treatments are to be characterized. In particular, the efficacy assessment of disinfection, including all technologies (e.g. by heat, chlorine, ozone, UV light, and so on), requires the application of representative and reliable indicators for viability and especially infectivity, often supplemented by selected reference pathogens. The assessment is historically based on cultivation methods, the considered *lege artis* gold standard, especially when disinfection processes and log-reduction targets are to be monitored, validated or verified. For example, a recent European Union regulation requires the cultivation-based validation monitoring of reclaimed water for agricultural irrigation (class A) using *E. coli*, somatic coliphages and *C. perfringens* spores, with defined performance targets of ≥5, ≥6, and ≥4 log_10_ reductions within the treatment chain, respectively (European Union 2020).

#### Generating viability- and infectious status information by molecular tools

In addition to cultivation-based enumeration, cultivation-independent, molecular strategies for viability- and infectious status analysis are also increasingly applied in research. For prokaryotes, a vast array of different techniques, including RNA-based methods (rRNA, messenger RNA), membrane integrity (e.g. viability stains, viability PCR), cellular metabolism (e.g. ATP, respiration, isotope labelling), protein-based methods (e.g. BONCAT), and microcalorimetry, have been suggested within the broad field of microbial ecology (Emerson et al. 2017). However, the delineation of dead versus viable microbial cells is complex and still under debate (Davey 2011, Kirschner et al. 2021). There is consensus that living microbial cells should have, (i) intact functional cell membranes, (ii) intact cellular- and energy metabolism, and (iii) the capability to reproduce (i.e. intact transcription/translation mechanisms). Straightforward determination strategies frequently address only one of these aspects of microbial viability (e.g. ‘live/dead’ protocols), leaving room for uncertainty (Emerson et al. 2017). Thus, (more time-consuming) multiple criteria are to be applied simultaneously, if precise viability characterization of the target microbiota is required (Kirschner et al. 2021). Detection of infectious viruses is equally challenging, and no single method is available to detect all infectious viruses in water (Gerba et al. 2018). At least three criteria must be fulfilled for infectious viruses, (i) sufficient genomic integrity to produce the required proteins for replication and to provide an accurate genetic template for subsequent generations, (ii) protection of the genome from degradation, and, (iii) the ability of the virus to recognize and infect the host cell (Pecson et al. 2009, Gerba et al. 2018).

Viability PCR and a similar approach, ET-qPCR, were introduced to the field of GFPD more than a decade ago (Bae and Wuertz 2009, Pecson et al. 2009) and have been increasingly applied in HRWM research in recent years. The original idea of applying viability PCR to bacterial MST markers was to gain information on recent faecal pollution events in water resources (Bae and Wuertz 2012, 2015). Viability PCR relies on the pretreatment of the sample with an intercalating dye, PMA, that penetrates cells with impaired membranes and prevents PCR-based amplification (Nocker et al. 2006), thus allowing the selective detection of cells with an intact membrane. Virus capsid integrity may also be assessed using the same principles (reviewed in Leifels et al. 2021) or using ET-PCR (Pecson et al. 2009). However, several authors note challenges related to conditions of procedure confounding the results and emphasize that experimental conditions need to be optimized and validated for the microorganism under investigation (Fittipaldi et al. 2012, Lazou et al. 2019, Leifels et al. 2021). The application of viability PCR now extends to the assessment of microorganism attenuation during treatment processes (section ‘*Application 4*’).

In summary, molecular tools to generate information on viability and infectious state constitute a novel and innovative area of research in GFPD. Relatively little experience exists in comparison to traditional PCR and qPCR analysis (section ‘*Application 4*’). Many challenges are still associated with their application, such as problems with methodical reproducibility, cross-reaction with background- or free nucleic acids, selection of optimal reagents, and experimental conditions and protocols (Gerba et al. 2018, Codony et al. 2020). Furthermore, the success of these methods often depends on the particular mechanism of inactivation (e.g. chemical vs. physical agents). Nonetheless, further development activities in the future will likely open new windows of opportunity in HRWM as well as in complementing cultivation-based standards. In addition, many potential areas within the range of these available molecular tools have not yet been exploited (Emerson et al. 2017). For example, and in contrast with viability PCR applications, RNA-based methods have only very rarely been applied and evaluated in GFPD (Pitkänen et al. 2013). As successfully demonstrated in other fields of environmental microbiology, RNA analysis may significantly contribute to information on the activity status of microbial populations (Gourse et al. 1996, Amann and Ludwig 2000, Deutscher 2006).

#### Sensitivity of environmental detection of nucleic acid targets

A common narrative is that molecular DNA/RNA diagnostics are highly specific and sensitive. This may be true for theoretical considerations. For ‘real world’ applications, this dictum, especially in relation to sensitivity, must be considered in the context of the overall analytical measurement challenge (Wintzingerode et al. 1997). For example, an optimally designed qPCR test should be able to detect, in theory, one target molecule of DNA/RNA, if present in a single reaction unit. However, as target molecules follow a stochastic distribution during analyte dilution for parallel analysis, the assay limit of detection (aLOD) cannot be less than three target molecules for a 95% detection probability per qPCR analysis, even with perfect PCR kinetics (Bustin et al. 2009). However, overall considerations require whole chain analysis (WCA), including sampling, recovered sampling volume, filtration- and enrichment-, nucleic acid extraction-, and purification efficacies, and finally, the amount of nucleic acid analysed (Table S2, Supporting Information). The resulting overall WCA sensitivity, reported for instance as the sample limit of detections (sLOD), can be quite elevated (Domingo et al. 2007). To illustrate, sLOD or alternative estimates on WCA sensitivity for qPCR DNA/RNA target enumeration were reported to be in the range of log_10_ 1.5–3.9 genetic targets per 100 ml sample (Pitkänen et al. 2013).

Selected genetic targets for GFPD often target highly abundant intestinal bacterial and viral populations as occurring in faecal excreta or wastewater, to compensate for the abovementioned WCA sensitivity issues. This fundamental design criterion is achieved by almost all top performing qPCR assays of genetic faecal markers (Layton et al. 2013, Reischer et al. 2013, Green et al. 2014b, Mayer et al. 2018, Sabar et al. 2022). Less abundant intestinal targets, such as traditional *E. coli* or enterococci (Farnleitner et al. 2010) can still be detected using genetic methods, if faecal pollution levels are elevated, as frequently observed for surface waters under communal and agricultural influence. However, in situations with low to very low faecal pollution levels, such as groundwater and drinking water resources, the sensitivity issues of genetic faecal markers can be very limiting. High-volume sampling, specific enrichment or alternative amplification systems may bring improved sensitivity and thus extend the possibilities of GFDP to such situations (Min and Baeumner 2002, Heijnen and Medema 2009, Rhodes et al. 2011, Liu et al. 2012).

In scenarios of low faecal pollution levels, it is common for a large proportion (>50%) of measurements to be below a GFPD method limit of quantification. For these censored data, the true genetic target concentration cannot be firmly established and can represent a significant source of bias in downstream statistical analyses. While it may be convenient to ignore censored data, these measurements offer important information. As a result, there is a growing interest in the development and use of statistical methods that can responsibly incorporate censored data into concentration estimates, hypotheses tests, regressions, and other analyses to help minimize potential bias and maximize faecal pollution trend insights. For example, Cao et al. (2018) developed a qPCR censored data faecal score approach to estimate a weighted-average genetic marker concentration from a defined group of samples using all measurements (e.g. nondetection, below the limit of quantification, or within the range of quantification). Additional research is needed to further advance censored data analysis methodologies custom designed for GFPD applications.

HTS applications as identified in our literature analysis (section ‘*Outcomes of the systematic study design analysis*’), face challenges in addition to WCA. In fact, the achievable sensitivity of 16S AmpSeq applications, applying general primers for broad taxonomic detection, such as kingdom and phylum level, strongly depends on the relative abundance of faecal pollution-associated intestinal microbiota compared to nonfaecal pollution associated microbiota (i.e. environmental ‘background microbiome’). Water resources, showing low to moderate faecal pollution levels and abundant aquatic microbiomes (e.g. 10^9^–10^11^ cells/l for lakes or rivers; Kirschner et al. 2004, Velimirov et al. 2011), become problematic, even when applying high amplicon sequencing-depth (Vierheilig et al. 2015). Consequently, identified studies have most frequently focused on water resources with significant municipal and agricultural faecal pollution levels (section ‘*In-depth review of the application areas of genetic faecal pollution diagnostic through case studies*’).

### GFPD (MST) application frame: status quo and research needs

#### Cutting-edge solutions require in-depth expert knowledge

The ability of GFPD methods to detect (is there a pollution problem?), quantify (what is the extent of pollution?), and allocate (what are the sources of pollution?) faecal pollution in water and water resources has undoubtedly revolutionized this area of HRWM research during the last two decades (Malakoff 2002). However, the application of general and host-associated faecal markers to generate accurate information on the responsible faecal pollution sources is not trivial. For example, the available genetic faecal marker targets as well as their quantification systems, differ in pollution source abundance and environmental persistence. Therefore, differences in these characteristics may severely compromise or prevent meaningful interpretation of results. Box 1 (upper panel, nonoptimal parameter setup) shows a hypothetical MST situation to illustrate the confusing effects that differential abundance and persistence of MST markers can impose for correct indication. Quantitative comparisons of MST results, or the more complex task of source apportionment (i.e. computation of faecal loads from the various sources), solely based on qPCR results, may therefore, only be achievable for a limited ‘diagnostic space’ (see the examples *t*0, *t*1, and *t*2 in Box 1, and ‘*A toolbox approach with case-dependent selection criteria*’ below). Having sound expert knowledge on the potentials and limits of GFPD is thus an essential prerequisite for correct application of GFPD in the field.

Box 1: Microbial source tracking markers: diagnostic scenarios

**Figure:**
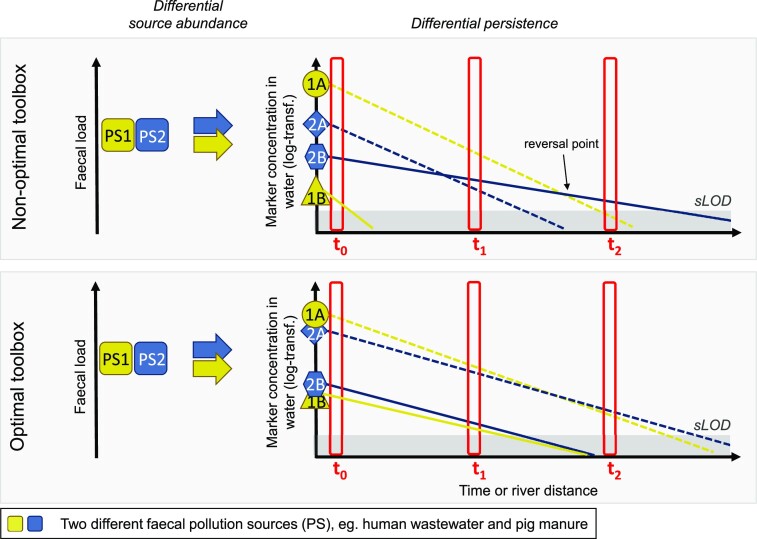

Simple hypothetical MST situation with two different point sources of pollution [e.g. human wastewater and animal (pig) manure] of equivalent discharge and contamination load for a small river. For reasons of simplicity, only dilution at the time of contamination (*t*0) and decay of the MST markers is considered (i.e. batch-reactor system with complete mixing and no sedimentation). Three time slots (*t*0, *t*1, and *t*2) are chosen to illustrate the different ‘diagnostic windows’ of MST indications at the given detection limit (sample limit of detection, sLOD).

*Nonoptimal toolbox*. All four applied MST markers show different abundance in their respective faecal excreta and persistence in the water body. At *t*0, all four MST markers allow correct qualitative detection of both sources (differential persistence insignificant). Due to the differential abundances of MST markers, no direct estimation on the relative importance of PS1/PS2 is possible. However, mathematical corrections of concentration differences in excreta would make this possible. At *t*1, MST marker 1B leads to false negative detection of PS1, due to differential persistence. Even in the case of accounting for differential abundance, only MST markers 1A and 2A can be used to estimate the relative importance of pollution of PS1/PS2 thanks to their similar persistence. At *t*2, only PS2 is detectable by MST marker 2B, thus the diagnosis would miss PS1 (false negative detection at the given sLOD).

*Optimal toolbox*. Both selected pairs of MST markers show comparable pollution source abundance in faecal excreta and persistence in the water body. The MST markers pair 1A–2A allow the estimation of the relative contribution of PS1 and PS2 at all times (*t*0–*t*2). Due to lower source abundance, the MST marker pair 1B–2B only allow detection and comparison at *t*0 and *t*1, but not at *t*2.

#### A toolbox approach with case-dependent selection criteria

No method comes without limitations, and no single method can have a universal application. Each genetic faecal parameter has specific biological–diagnostic and technical–analytical attributes (Table 2; Table S1, Supporting Information). The selection of diagnostic tools, as well as the chosen field investigation strategy, should therefore, be designed to best suit the given faecal pollution problem (Schoen et al. 2020), including a sound knowledge of the catchment characteristics and hydrological regime (Reischer et al. 2008). A basic catchment survey or pollution source profile can substantially improve the understanding of the situation and guide the selection of GFPD parameters and methods with appropriate performance characteristics (Reischer et al. 2011, Derx et al. 2023).

In addition to persistence, it is equally essential for MST to have appropriate (binary) faecal sensitivity and specificity of the selected genetic marker (Table 2). The minimum acceptable levels of faecal sensitivity and specificity depend on the faecal pollution scenario under investigation (such as the relative abundance of the diagnosed faecal pollution sources). These levels can be determined through statistical considerations or catchment-based scenario simulations (Kildare et al. 2007, Reischer et al. 2011, Derx et al. 2021). A well-selected combination of markers, along with an algorithm that considers the sensitivity and specificity characteristics of the markers, enables more confident source identification compared to an individual marker (Ballesté et al. 2020). Faecal specificity is also important for general faecal markers (should be absent in a pristine environment, Table 2) and, in analogy with MST markers, should be evaluated in the studied catchments (Vierheilig et al. 2012). There are significant knowledge gaps regarding the mobility of indicator microorganisms and viruses detected by GFPD (Table 2). Mobility can be an essential factor in almost any natural and technical aquatic compartment. For example, mobility may codetermine the fate of MST markers, (i) in deposited fresh cow pats on pastures (e.g. activation tendency and run-off during rainfall; Devane et al. 2022); (ii) during wastewater treatment (e.g. attachment to sewage sludge fraction or dispersion in the water phase; Wang et al. 2023); (iii) in surface water transport processes, such as river water (e.g. attachment to settling particles and sedimentation or transport in suspended fraction; Fauvel et al. 2017); or (iv) during river bank filtration (e.g. straining or attachment in the aquifer or aquifer transport; Wang et al. 2022). Mobility characteristics are often complex, as they are potentially influenced by physical, chemical and biological processes, depending on the aquatic scenario. Finally, the resistance of genetic markers to technical treatment is an additional important biological–diagnostic attribute (Table 2). Different resistance is expected for cultivation-based parameters than for DNA/RNA-based parameters (section ‘*Generating viability- and infectious status information*’). For instance, in contrast to cultivation-based FIO concentrations, almost no reduction was observed for qPCR-based prokaryotic MST markers during UV-treatment of wastewater and drinking water (Steinbacher et al. 2021).

#### Prokaryotic targets dominate, but the importance of viral targets increases

**Figure 14.**
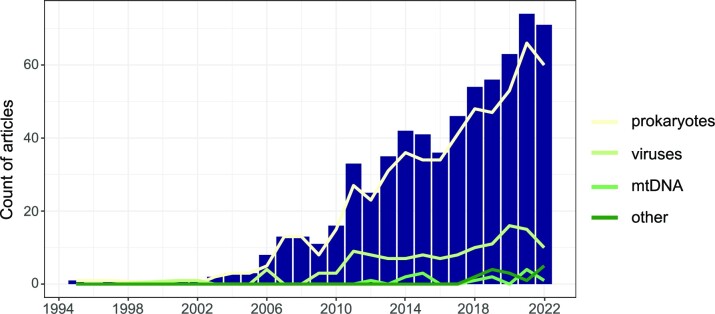
Timeseries of microorganisms targeted by genetic methods, with the bar chart of all papers per year in the background (*n*_article_ = 649). mtDNA indicates host mitochondrial DNA.

Key biological elements in the current state-of-the-art toolbox are the various general- and host-associated faecal markers, quantified by qPCR or dPCR assays (section ‘*Background information on genetic targets and methods: a historical overview*’). The systematic literature analysis revealed that prokaryotic faecal markers have dominated GFPD up to now (Fig. 14). However, viral faecal markers have seen an increase in the past 10 years, while mitochondrial markers have been applied to a far lesser extent (Fig. 14). The combined use of selected MST markers, with adequate performance characteristics, hold promise for detecting and allocate faecal pollution with increased confidence, even under challenging faecal pollution scenarios (e.g. undiluted vs. diluted, fresh versus aged, untreated vs. treated faecal pollution). In this respect, complementing prokaryotic GFPD applications with viral faecal markers can be especially important to account for the increased persistence, resistance and mobility characteristics of such types of intestinal contaminants (‘*Application 4: Microorganisms attenuation during treatment*’ and ‘*Application 5: Estimating of infection and health risk*’). Cultivation-based FIO (see discussion ‘hybrid application’ below), pathogen detection and antibiotic resistance analysis complements the current array of biological elements in GFPD (Fig. 5).

The GFPD toolbox is steadily growing, although now at a slower pace than during the first pioneering decade of the new millennium (Fig. 3, number of establishment/application studies). New genetic faecal markers and/or improved detection systems are, without any doubt, essential for the further development of the discipline. However, it should be kept in mind that providing detailed information on their environmental behaviour and application characteristics (Table 2; Tables S1 and S2, Supporting Information) is equally important, if not more, to successfully implement them in HRWM research (Fig. 13). There is a disparity between the availability of genetic markers and detection systems compared to the availability of information on their biological–diagnostic characteristics and on their applicability in different types of water resources and subcompartments (under given biotic and abiotic conditions, e.g. (Boehm et al. 2019, Korajkic et al. 2019, Lu and Imlay 2021)).

#### Integrated data analysis and modelling

The inclusion of other microbiological and environmental parameters into the study design can greatly enhance the information gained from GFPD investigations. Remarkably, almost two-thirds of the identified source tracking studies (single and multiple sources) simultaneously applied genetic MST-marker qPCR quantification and traditional cultivation-based enumeration of FIOs (Fig. 3), determined by standardized parameters such as those for *E. coli* (ISO 1998a, 2012, 2014, 2018) or intestinal enterococci (ISO 1998b, 2000). The need to determine the causes responsible for faecal pollution in water obviously promotes this most popular ‘hybrid application’ (sections ‘*Application 2*’ and ‘*Application 3*’). In addition, data on pathogen occurrence and physicochemical water quality were used to complement the investigation, although to a far lesser extent. In contrast, the identified GFPD studies hardly utilized information on hydrology, meteorology, land use or epidemiology for statistical data analysis (Fig. 5). This is contrary to expectations, as environmental data, such as data on catchment hydrology and land use (GIS, mapping), have proven essential for an improved application, understanding and interpretation of GFPD in water quality research (Reischer et al. 2008, Peed et al. 2011, Bambic et al. 2015, Verhougstraete et al. 2015, Frick et al. 2020, Green et al. 2021, Diedrich et al. 2023). Without a doubt, there is significant potential to better utilize and integrate environmental data in GFPD analysis in future HRWM research (Fig. 13).

Data from GFPD, together with FIO and pathogens, are increasingly used in modelling and simulation. Potential areas of interest include all issues and scales of HRWM research (ranging from faecal marker persistence/dilution models to catchment-based source/sink transport simulations) as well as application types (such as faecal pollution, MST, treatment, and infection- and health risk assessment) as covered in this literature analysis (Dorner et al. 2006, Sokolova et al. 2012, Boehm et al. 2015, Pascual-Benito et al. 2020, Derx et al. 2021). To highlight the importance, modelling becomes essential, e.g. to estimate the required microbial/viral log reduction targets for wastewater or drinking water treatment or to determine the appropriate setback distances during riverbank filtration. Importantly, modelling and simulations also allow the assessment of future scenarios and even the prediction of the management measures that will be required considering future climate and global change phenomena (Demeter et al. 2021). One of the big challenges of modelling and simulation in health-related water quality research and GFPD is to provide all the data and data collections required (Fig. 13).

## Conclusions

The tools and approaches developed for GFPD have revolutionized HRWM research in the last two decades in terms of faecal pollution detection and microbial source tracking, the current core areas of application. Together with nucleic acid extract biobanking, GFPD represents a new level of methodological possibilities in health-related water quality research in the 21st century, even in remote or less developed regions.
GFPD is ready to expand to many other application areas within and outside the field of HRWM. For instance, it will further gain importance in infection and health risk assessment (e.g. recreational water quality monitoring) and will increasingly support the evaluation and verification of water treatment and disinfection processes, in combination with standardized treatment indicators and cultivation-based enumeration.
The COVID-19 pandemic gave a strong boost to the field of wastewater surveillance. Wastewater surveillance for SARS-CoV-2 is currently transforming into a global early warning disease monitoring system. GFPD will likely increasingly support wastewater surveillance in data generation, pollution source characterization, normalization, and quality assurance. Since both ‘sister’ disciplines use the same molecular biological framework and infrastructure, potential synergies are significant. In general, GFPD has the potential to support any environmental global infectious disease surveillance system, covering human and other animal populations.
As demonstrated by the many identified studies, internationally accepted, cultivation-based water quality parameters, such as *E. coli* or intestinal enterococci, can be effectively complemented with GFPD, thus significantly expanding the methodical possibilities in water quality monitoring and management, when needed (e.g. MST to trace the origin of cultivation-based FIO). GFPD constitutes a toolbox approach. Tailor-made scientific investigation and monitoring solutions can be rapidly established by experts.
The current century is ‘the Century of Life Sciences’, especially considering how molecular biology and bioinformatics rapidly transform health sciences and medicine. It is also the era of information technology, artificial intelligence, and automatization. These driving forces will certainly promote further innovation within genetic faecal pollution detection. Many technological breakthroughs are expected.
From science to practice. The water management sector increasingly needs the tools and approaches offered by GFD to solve future challenges (e.g. challenges related to SDG6). The translation of such tools to practice has to be paralleled by standardization efforts. While some countries have already started such activities (e.g. three assays are standardized in the USA), international standards are still lacking. These needs will have to be defined by the water management sector and translated to future GFPD guidelines and standards by global panels of experts.
This meta-analysis provides the scientific status quo of the field of GFPD. It should promote further research to advance the scientific field and serve as a condensed information source for the wider audience, including microbiologists, water hygienists, water management professionals, and public health experts.

## Supplementary Material

fuad028_Supplemental_Files
