# Supplementary material for: Have genetic targets for faecal pollution diagnostics and source tracking revolutionized water quality analysis yet?
Source: FEMS Microbiol Rev. 2023 Jun 7;47(4):fuad028. doi: 10.1093/femsre/fuad028 (PMC10368376; doi:10.1093/femsre/fuad028)
Supplement: fuad028_Supplemental_Files [file fuad028_supplemental_files.zip › Demeter et al GFPD review Suppl Information.docx]

***Have genetic targets for faecal pollution diagnostics and source tracking revolutionised water quality analysis yet?***

**Supplementary Information**

*Katalin Demeter^1,#^, Rita Linke^1,#^, Elisenda Ballesté^2^, Georg Reischer^1,#^, René E. Mayer^1,4,#^, Julia Vierheilig^3,#^, Claudia Kolm^4,#^, Margaret E. Stevenson^5,#^, Julia Derx^5,#^, Alexander K.T. Kirschner^4,6,#^, Regina Sommer^6,#^, Orin C. Shanks^7^, Anicet R. Blanch^2^, Joan Rose^8^, Warish Ahmed^9^, Andreas H. Farnleitner^1,4,#,*^*

^1^ Institute of Chemical, Environmental and Bioscience Engineering, TU Wien, Austria;

^2^ Department of Genetics, Microbiology and Statistics, University of Barcelona, Spain

^3^ Institute of Water Quality and Resource Management, TU Wien, Austria;

^4^ Karl Landsteiner University of Health Sciences, Krems a/d Donau, Austria;

^5^ Institute of Hydraulic Engineering and Water Resources Management, TU Wien, Austria;

^6^ Institute for Hygiene and Applied Immunology, CePII, Medical University of Vienna, Austria;

^7^ U.S. Environmental Protection Agency, Office of Research and Development, Cincinnati, USA;

^8^ Michigan State University, East Lansing, USA;

^9^ Dutton Park, Ecosciences Precinct, Commonwealth Scientific and Industrial Research Organisation, Queensland, Australia;

# Interuniversity Cooperation Centre Water & Health, Austria

* corresponding author

**Table S1.** Overview on essential analytical-technical performance criteria for the isolated step of the polymerase chain reaction (PCR). Most of the shown attributes can be further divided into sub-characteristics. Various methods and specification metrics have been suggested for determination.

| **Characteristic** | **Basic Definition** | **Remarks** | **Methods / Metrics** |
| --- | --- | --- | --- |
| **Assay Limit of Detection (LOD)** | The number of the minimal target copies detectable in the analyte (nucleic acid extract) by PCR for a given probability.  **Synonyms:** analytical limit of detection (aLOD), method detection limit (MDL) | The LOD is a probabilistic parameter which is usually estimated as the target concentration at which there is a 95% chance of detection. According to the Poisson distribution, the LOD_95%_ for PCR cannot be lower than at least three target copies in a reaction. | The LOD can be determined using known quantities of a target, such as a plasmid containing the genetic marker of interest (Domingo *et al.*, 2007, Bustin *et al.*, 2009, Forootan *et al.*, 2017, Borchardt *et al.*, 2021). |
| **Assay Limit of quantification (LOQ)** | The number of target copies in the analyte (nucleic acid extract) that can be quantified with a certain precision.  **Synonyms:** analytical limit of quantification (aLOQ) | The LOQ can be equal to the LOD, but can never be lower than the LOD. | It is common practice to report the LOQ as a coefficient of variation (CV; e.g., < 25%). E.g. (Forootan *et al.*, 2017, Kralik & Ricchi, 2017, Ruiz-Villalba *et al.*, 2021). |
| **Precision** | The random deviation from repeated measurements.  Precision, as the technical variation, should be distinguished from biological variation. | Many factors can influence variation in PCR results, e.g., temperature differences affecting the completion of annealing and/or denaturation, template concentration, analyst pipetting, among others. | Precision varies with concentration. Intra-assay variation can be displayed as standard deviation or as confidence interval based on repeated testing of a sample (Bustin *et al.*, 2009, Kubista, 2014, Tellinghuisen & Spiess, 2014, Kralik & Ricchi, 2017). |
| **Accuracy** | The degree of agreement of measurements with the actual or true amount in the theoretical absence of variability. | Accuracy refers to the difference between experimentally measured and actual concentrations. | Accuracy can be presented as fold changes or copy number estimates (Bustin *et al.*, 2009, Kubista, 2014, Tellinghuisen & Spiess, 2014, Kralik & Ricchi, 2017). |
| **Linear dynamic range** | The maximum range over which an accurate and reliable measurement can be made.  **Synonyms:** range of quantification | The linear dynamic range is typically expressed as a linear regression using R^2^ to evaluate the quality of fit. | A linear dynamic range should cover three or more orders of magnitude depending on PCR platform (e.g., qPCR, dPCR) and ideally should exhibit an R^2^ ≥ 0.980 (Bustin *et al.*, 2009, Kubista, 2014, Tellinghuisen & Spiess, 2014, Kralik & Ricchi, 2017). |
| **Amplification efficiency** | The fraction of target molecules that are copied in one PCR amplification cycle. | Amplification efficiency is routinely determined from the slope of a calibration curve. | Amplification efficiency is typically expressed as a percent and can be determined from the slope of the log-linear part of a calibration curve (Bustin *et al.*, 2009, Kubista, 2014, Tellinghuisen & Spiess, 2014, Kralik & Ricchi, 2017). |

**Table S2.** Overview of essential analytical-technical performance criteria including all individual steps of the whole chain of analysis (WCA), such as sample collection, filtration, extraction, and detection/quantification. Various methods and specification metrics have been suggested for determination.

| **Characteristic** | **Basic Definition** | **Remarks** | **Methods / Metrics** |
| --- | --- | --- | --- |
| **Sample Limit of Detection (SLOD)** | The number of the minimal detectable target copies in the original sample for a given probability (e.g., SLOD 95%), considering all sample processing steps.  **Synonyms:** PLOD (process limit of detection), eLOD (equivalent limit of detection ) | The SLOD can be determined on an individual sample basis or for a defined sample type (e.g., wastewater, surface water, etc.) associated with a defined method protocol.  The SLOD can be affected by losses during sample preparation (e.g., concentration, nucleic acid extraction, purification, reverse transcription, inhibitory matrix effects (e.g. Linke *et al.* 2021), and the individual LOD for respective PCR reaction). | SLOD can be determined in analogous way as LOD, except that the original sample material or spiked material of the same/similar matrix are used. E.g., (Reischer *et al.*, 2006, Domingo *et al.*, 2007, Rajal *et al.*, 2007, Pitkänen *et al.*, 2013, Symonds *et al.*, 2016, Kolm *et al.*, 2019, Barrios *et al.*, 2021, Ahmed *et al.*, 2022)  There are no uniform metrics for SLOD. |
| **Threshold of Detection (ToD)** | The number of the minimal detectable target copies in the original sample assuming no losses along the WCA. | The ToD is a simple and practicable metric to estimate the SLOD in case the SLOD cannot be determined. | This metric was established for springs and other drinking water resources (Reischer *et al.*, 2007, Reischer *et al.*, 2008), and later applied for many other matrices (Kirschner *et al.*, 2017, Mayer *et al.*, 2018, Derx *et al.*, 2023).  The ToD is given as marker equivalents per volume (ME/volume) instead of copies per volume, since the actual filtration and extraction efficiencies remain unknown (set statistically to 1). |
| **Sample Limit of quantification (SLOQ)** | The number of target copies in a sample that can be quantified with a certain precision considering the WCA.  **Synonym:** PLOQ (process limit of quantification) | The SLOQ can be equal to SLOD, but can never be lower than the SLOD. | SLOQ is determined in the same way as LOQ, except that dilutions of the sample material or spiked material of the same matrix are typically used. E.g., (Symonds *et al.*, 2016). |
| **Sample Amount** | Amount of sample (e.g., volume, mass) collected and used for subsequent processing. | The required amount depends on the expected target concentration in the sample as well as the efficiency each step in the WCA. | WCA results should be reported up to the original sample amount used for testing. Downscaling reported results is possible, but upscaling is not recommended. |
| **Process Control** | Monitors the efficiency of one or more steps of the WCA and allows conclusions on potential measurement bias (e.g., matrix interference, amplification inhibition, etc.). | The choice of process control often depends on the target organism to be detected/quantified and on the application. | Process control methods and metrics vary by research question. E.g., (Domingo *et al.*, 2007, Stoeckel *et al.*, 2009, Green *et al.*, 2012). |
| **DNA storage and preservation (Biobanking)** | Nucleic acid preparations can be stored for an extended period of time. This offers the possibility to perform further investigations for an extended period of time. | There are many options for storage of nucleic acids such as lyophilized (freeze dried), suspension in various buffers, binding to assorted filter materials, among others. Currently, there is no consensus on the ideal storage practice nor the maximum time period. | Short-term storage of nucleic acid preparations can be done at -20°C. Long-term storage of nucleic acid preparations is usually done at -80°C or in liquid nitrogen (-196°C). E.g., (De Paoli, 2005, Jackson *et al.*, 2011, Cary & Fierer, 2014). |

**References**

Ahmed W, Bivins A, Metcalfe S, Smith WJM, Verbyla ME, Symonds EM & Simpson SL (2022) Evaluation of process limit of detection and quantification variation of SARS-CoV-2 RT-qPCR and RT-dPCR assays for wastewater surveillance. *Water Res* **213**: 118132.

Barrios ME, Díaz SM, Torres C, Costamagna DM, Blanco Fernández MD & Mbayed VA (2021) Dynamics of SARS-CoV-2 in wastewater in three districts of the Buenos Aires metropolitan region, Argentina, throughout nine months of surveillance: A pilot study. *Science of The Total Environment* **800**: 149578.

Borchardt MA, Stokdyk JP, Kieke BA, Muldoon MA, Spencer SK, Firnstahl AD, Bonness DE, Hunt RJ & Burch TR (2021) Sources and Risk Factors for Nitrate and Microbial Contamination of Private Household Wells in the Fractured Dolomite Aquifer of Northeastern Wisconsin. *Environmental Health Perspectives* **129**.

Bustin SA, Benes V, Garson JA*, et al.* (2009) The MIQE Guidelines: Minimum Information for Publication of Quantitative Real-Time PCR Experiments. *Clin Chem* **55**: 611-622.

Cary SC & Fierer N (2014) The importance of sample archiving in microbial ecology. *Nature reviews Microbiology* **12**: 789-790.

De Paoli P (2005) Bio-banking in microbiology: from sample collection to epidemiology, diagnosis and research. *FEMS microbiology reviews* **29**: 897-910.

Derx J, Kılıç HS, Linke R*, et al.* (2023) Probabilistic fecal pollution source profiling and microbial source tracking for an urban river catchment. *Science of The Total Environment* **857**: 159533.

Domingo JWS, Bambic DG, Edge TA & Wuertz S (2007) Quo vadis source tracking? Towards a strategic framework for environmental monitoring of fecal pollution. *Water Research* **41**: 3539-3552.

Forootan A, Sjöback R, Björkman J, Sjögreen B, Linz L & Kubista M (2017) Methods to determine limit of detection and limit of quantification in quantitative real-time PCR (qPCR). *Biomolecular detection and quantification* **12**: 1-6.

Green HC, Dick LK, Gilpin B, Samadpour M & Field KG (2012) Genetic Markers for Rapid PCR-Based Identification of Gull, Canada Goose, Duck, and Chicken Fecal Contamination in Water. *Applied and Environmental Microbiology* **78**: 503-510.

Jackson J, Laikre L, Baker C & Kendall K (2011) Guidelines for collecting and maintaining archives for genetic monitoring. *Conservation Genetics Resources* **4**.

Kirschner AKT, Reischer GH, Jakwerth S*, et al.* (2017) Multiparametric monitoring of microbial faecal pollution reveals the dominance of human contamination along the whole Danube River. *Water Research* **124**: 543-555.

Kolm C, Martzy R, Führer M, Mach RL, Krska R, Baumgartner S, Farnleitner AH & Reischer GH (2019) Detection of a microbial source tracking marker by isothermal helicase-dependent amplification and a nucleic acid lateral-flow strip test. *Scientific Reports* **9**: 393.

Kralik P & Ricchi M (2017) A Basic Guide to Real Time PCR in Microbial Diagnostics: Definitions, Parameters, and Everything. *Frontiers in Microbiology* **8**.

Kubista M (2014) Prime time for qPCR - Raising the quality bar. *European Pharmaceutical Review* **19**: 63-67.

Mayer RE, Reischer GH, Ixenmaier SK*, et al.* (2018) Global Distribution of Human-Associated Fecal Genetic Markers in Reference Samples from Six Continents. *Environmental Science & Technology* **52**: 5076-5084.

Pitkänen T, Ryu H, Elk M, Hokajärvi AM, Siponen S, Vepsäläinen A, Räsänen P & Santo Domingo JW (2013) Detection of fecal bacteria and source tracking identifiers in environmental waters using rRNA-based RT-qPCR and rDNA-based qPCR assays. *Environ Sci Technol* **47**: 13611-13620.

Rajal VB, McSwain BS, Thompson DE, Leutenegger CM & Wuertz S (2007) Molecular quantitative analysis of human viruses in California stormwater. *Water Research* **41**: 4287-4298.

Reischer GH, Kasper DC, Steinborn R, Mach RL & Farnleitner AH (2006) Quantitative PCR method for sensitive detection of ruminant fecal pollution in freshwater and evaluation of this method in alpine karstic regions. *Applied and environmental microbiology* **72**: 5610-5614.

Reischer GH, Kasper DC, Steinborn R, Farnleitner AH & Mach RL (2007) A quantitative real-time PCR assay for the highly sensitive and specific detection of human faecal influence in spring water from a large alpine catchment area. *Letters in Applied Microbiology* **44**: 351-356.

Reischer GH, Haider JM, Sommer R, Stadler H, Keiblinger KM, Hornek R, Zerobin W, Mach RL & Farnleitner AH (2008) Quantitative microbial faecal source tracking with sampling guided by hydrological catchment dynamics. *Environmental Microbiology* **10**: 2598-2608.

Ruiz-Villalba A, Ruijter JM & van den Hoff MJB (2021) Use and Misuse of C(q) in qPCR Data Analysis and Reporting. *Life (Basel, Switzerland)* **11**.

Stoeckel DM, Stelzer EA & Dick LK (2009) Evaluation of two spike-and-recovery controls for assessment of extraction efficiency in microbial source tracking studies. *Water Res* **43**: 4820-4827.

Symonds EM, Sinigalliano C, Gidley M, Ahmed W, McQuaig-Ulrich SM & Breitbart M (2016) Faecal pollution along the southeastern coast of Florida and insight into the use of pepper mild mottle virus as an indicator. *Journal of Applied Microbiology* **121**: 1469-1481.

Tellinghuisen J & Spiess AN (2014) Comparing real-time quantitative polymerase chain reaction analysis methods for precision, linearity, and accuracy of estimating amplification efficiency. *Anal Biochem* **449**: 76-82.
